# Goofballing of Opioid and Methamphetamine: The Science Behind the Deadly Cocktail

**DOI:** 10.3389/fphar.2022.859563

**Published:** 2022-04-07

**Authors:** Hanis Mohammad Hazani, Isa Naina Mohamed, Mustapha Muzaimi, Wael Mohamed, Mohamad Fairuz Yahaya, Seong Lin Teoh, Rashidi Mohamed Pakri Mohamed, Mohd Fadzli Mohamad Isa, Sundus Mansoor Abdulrahman, Ravi Ramadah, Mohammad Rahim Kamaluddin, Jaya Kumar

**Affiliations:** ^1^ Department of Physiology, Faculty of Medicine, The National University of Malaysia, Cheras, Malaysia; ^2^ Department of Pharmacology, Faculty of Medicine, The National University of Malaysia, Cheras, Malaysia; ^3^ Department of Neurosciences, School of Medical Sciences, Universiti Sains Malaysia, Kota Bharu, Malaysia; ^4^ Basic Medical Science Department, Kulliyyah of Medicine, International Islamic University Malaysia, Kuantan, Malaysia; ^5^ Faculty of Medicine, Department of Clinical Pharmacology, Menoufia University, Shebin El-Kom, Egypt; ^6^ Department of Anatomy, Faculty of Medicine, National University of Malaysia, Cheras, Malaysia; ^7^ Department of Family Medicine, Faculty of Medicine, The National University of Malaysia, Cheras, Malaysia; ^8^ Department of Psychiatry and Mental Health, Hospital Kuala Lumpur, Kuala Lumpur, Malaysia; ^9^ Newcastle University Medicine Malaysia, Johor, Malaysia; ^10^ National Anti-Drugs Agency Malaysia, Selangor, Malaysia; ^11^ Centre for Research in Psychology and Human Well-Being, Faculty of Social Sciences and Humanities, The National University of Malaysia, Bangi, Malaysia

**Keywords:** addiction, dependence, polydrug, polysubstance, opioid, methamphetamine, abuse, goofball

## Abstract

Globally, millions of people suffer from various substance use disorders (SUD), including mono-and polydrug use of opioids and methamphetamine. Brain regions such as the cingulate cortex, infralimbic cortex, dorsal striatum, nucleus accumbens, basolateral and central amygdala have been shown to play important roles in addiction-related behavioral changes. Clinical and pre-clinical studies have characterized these brain regions and their corresponding neurochemical changes in numerous phases of drug dependence such as acute drug use, intoxication, craving, withdrawal, and relapse. At present, many studies have reported the individual effects of opioids and methamphetamine. However, little is known about their combined effects. Co-use of these drugs produces effects greater than either drug alone, where one decreases the side effects of the other, and the combination produces a prolonged intoxication period or a more desirable intoxication effect. An increasing number of studies have associated polydrug abuse with poorer treatment outcomes, drug-related deaths, and more severe psychopathologies. To date, the pharmacological treatment efficacy for polydrug abuse is vague, and still at the experimental stage. This present review discusses the human and animal behavioral, neuroanatomical, and neurochemical changes underlying both morphine and methamphetamine dependence separately, as well as its combination. This narrative review also delineates the recent advances in the pharmacotherapy of mono- and poly drug-use of opioids and methamphetamine at clinical and preclinical stages.

## Introduction

Dependence on drugs and alcohol is a serious worldwide problem from social, economic, and health perspectives (Pakri Mohamed et al., 2018; Das and Horton, 2019; Sontate et al., 2021). Globally, the number of methamphetamine and opiate users has continued to grow at an alarming rate despite numerous stringent drug abuse laws (Bach et al., 2020; Dezman et al., 2020). A recent report indicates a nearly four-fold increase in methamphetamine-related hospitalizations and a more than 10-fold increase in stimulant-related deaths (Winkelman et al., 2018; Ruhm, 2019) surpassing the overdose death rate of prescription opioids (Hedegaard et al., 2020). Likewise, the prevalence of opioid overdose and overdose-related deaths were also escalated in the past years (Stevens et al., 2017; Sivaraman et al., 2021). What’s more concerning is, almost half of psychostimulant use-related deaths involve opioids, and an increase in trend is also observed in opioid use related-deaths involving methamphetamine (Ihongbe and Masho, 2016; Lancet, 2018; Gladden et al., 2019; Kariisa et al., 2019), indicating a spike in polysubstance use (Palamar et al., 2018; Zuckermann et al., 2019; Compton et al., 2021). It is estimated that the global cost for the treatment of 4.5 million drug users is about $35 billion annually (INCB, 2013), which is accounted for only one in six drug users. If all of the dependent drug users were to seek treatment, it would cost an estimated 0.3–0.4% of the global gross domestic product ($200 billion) (INCB, 2013). The cost of untreated and continuing use is significantly higher than investment in treatment alone, research finds. Reports from the United States National Drug Intelligence Center (NDIC) indicate the drug-related healthcare cost includes both direct and indirect costs related to inpatient drug treatment, medical intervention such as emergency services, and research for prevention and treatment (NDIC, 2011).

Currently, one of the most well-researched treatment options for substance dependence is opioid dependence. Methadone maintenance therapy (MMT) has been employed as one of the harm reduction approaches to manage opiate addiction (Ali et al., 2018), with some reporting its efficacy in reducing high-risk behaviors (Zhang et al., 2019), and whereas some have argued that for long term treatment, MMT may not significantly improve the quality of life among patients (Teoh Bing Fei et al., 2016). MMT also requires lifelong commitments from drug users. Other drugs such as buprenorphine or buprenorphine-naloxone are mainly used in private settings due to the high cost, as a maintenance therapy (Vijay et al., 2015). Buprenorphine is an opioid agonist like methadone, whereas naloxone is a short-acting opioid antagonist commonly given by injection to reverse opioid overdoses (Webster et al., 2016). In several countries, buprenorphine or buprenorphine-naloxone combinations were injected illicitly by the majority of opioid users, increasing the incidences of opioid dependence (Yokell et al., 2011). Oral treatment of naltrexone for opioid dependence is ineffective due to poor treatment adherence (Minozzi et al., 2011). Naltrexone implant, on the other hand, has produced some positive results in the treatment of opioid or polydrug abuse (Kelty et al., 2019; Krupitsky et al., 2019). Nevertheless, the clinical efficacy of the implant in the long-term has not been reported and the potential opioid overdose associated with naltrexone implant has not been sufficiently explored (Saucier et al., 2018).

To date, there are no significantly convincing treatment outcomes in the pharmacotherapy of methamphetamine use disorder (MUD) (Morley KC. et al., 2017; Ballester et al., 2017). Systematic analysis of existing literature revealed some positive outcomes with dexamphetamine, methylphenidate, naltrexone, and topiramate, whereas anti-depressants, such as selective serotonin reuptake inhibitors, and tricyclic antidepressants were being the least effective in the management of MUD (Siefried et al., 2020). Individual clinical studies have reported efficacy in the use of buprenorphine (Ahmadi and Razeghian Jahromi, 2017; Ahmadi et al., 2019), N-acetylcysteine (Salehi, 2015), and methylphenidate (Rezaei et al., 2015) in reducing the craving score of methamphetamines, whereas some have reported lack of efficacy among drugs such as bupropion (Anderson et al., 2015), modafinil (Heinzerling et al., 2010; Anderson et al., 2012), varenicline (Briones et al., 2018) in methamphetamine dependence treatment.

Therefore, this present review discusses the human and animal behavioral and neurochemical changes underlying both morphine and methamphetamine dependence separately, as well as its combination. This review also delineates the recent advances in the pharmacotherapy of mono and poly drug-use of opioids and methamphetamine at clinical and preclinical stages.

## Opioid Use Disorder

Opioid abuse originates from over prescription for the patients’ pain relief, while the increasing availability of low-cost opioids also has exacerbated its potential for abuse (Darcq and Keiffer, 2018). Patients develop tolerance to the opioid’s analgesic effect after treatment over an extended period. Administration of opioids at a higher dose is used to overcome this tolerance, however, patients will then be vulnerable to severe side effects such as withdrawal symptoms, and the threat of respiratory depression (Hayhurst and Durieux, 2016). Worldwide, the prevalence of opioid use was the highest in North America (UN World Drug Report, 2021). Analysis of individual data from the United Kingdom, United States, Australia, Germany, and France revealed that almost 1 in 5 reported abuse and 1 in 4 individuals reported misuse of opioid analgesics obtained through a prescription (Morley KI. et al., 2017). Heroin, fentanyl and morphine were the most commonly used opioids amongst others which include methadone, buprenorphine, codeine, tramadol, oxycodone, and hydrocodone (UN World Drug Report, 2019). According to WHO estimates, there were approximately 115,000 casualties from opioid overdoses globally, and COVID-19 has further exacerbated the fatality rate (Centers for Disease Control and Prevention, 2020; UN World Drug Report, 2021).

Morphine abuse negatively affects the users once the addiction cycle is engaged due to the tolerance developed following prolonged use of morphine, which is defined as the need to increase the dose to achieve the same initial effect due to decreased analgesic efficacy (Dai et al., 2018). The Food and Drug Administration (FDA) defines a person is opioid-tolerant if the person has been receiving oral morphine 60 mg/day for 1 week, where different types of opioids have different durations such as transdermal fentanyl, oral oxycodone, oral hydromorphone, oral oxymorphone with 25 mg/h, 30 mg/day, 8 mg/day, 25 mg/day, respectively (Rabin et al., 2017). The users potentially succumb to dependence due to the severity of the withdrawal symptoms including abdominal pain, nausea, diarrhea, lacrimation, and generalized piloerection. In contrast to the drug pain-relieving effects, drug cessation in the morphine-dependent state results in the genesis of negative effects such as anxiety, agitation, and dysphoria (Verster et al., 2021). Psychological dependence on the other hand refers to the state of the patient where they are craving for the drug, to relieve its withdrawal symptoms, or for its gratifying effects (Jacobs, 1986). The withdrawal symptoms that are brought forth from abstinence lead to craving with disinhibition, leaving the user vulnerable to relapse (Kalant, 2010; Campbell et al., 2013). Moreover, there was heightened impulsivity and impaired strategic planning in opioid-dependent patients (Tolomeo et al., 2016), along with increased anhedonia (Kras et al., 2018; Kiluk et al., 2019). Withdrawal symptoms are a key driver behind continued abuse, and a barrier to opioid discontinuation (Pergolizzi et al., 2020).

Behavior parameters established using various models of abuse under controlled environmental and drug administration regimens mimic the psychological status of humans in the presence or absence of substances depending on the animal models (Kumar et al., 2013; Kumar et al., 2016; Iman et al., 2021). Likewise, in opioid dependence animal models, depressive-like behaviors are significant at 1 week after prolonged withdrawal where experiments showed that there was a decreasing level of social interaction and elevation in immobility time which reflects a state of lowered mood or depression-like behavior (Anraku et al., 2001). The social avoidance symptoms and emotional despair mirrored by these mice reflect depression (Jia et al., 2013). Anxiety is another prominent affective symptom that manifests during abstinence from chronic morphine administration. Animal studies have shown that there is a significant increase in anxiety-like behaviors in the elevated plus maze and light/dark box paradigms (Zhang et al., 2008; Buckman et al., 2009; Miladi-Gorji et al., 2012). Apart from that, another prominent withdrawal symptom that accompanies abstinence is impulsive behavior, which encapsulate poor inhibitory response control (impulsive action) and impulsive decision making (impulsive choice) where observations suggest that the opioid system plays a significant role in decision making (Pattij et al., 2009). Morphine exposure also increases motor impulsivity in animal models (Kieres et al., 2004; Colin et al., 2012; Moazen et al., 2018), as well as deficits in learning and memory (Iman et al., 2021).

In Europe, fentanyl abuse was related to over 250 fatalities, while in 2017 alone there have been 25 fatalities associated with fentanyl and its synthetics analogs such as carfentanil, butyryl fentanyl, fluorobutyrylfentanyl, furanylfentanyl, and alfentanil (European Monitoring Centre for Drug and Drug Addiction, 2018; Hikin et al., 2018). According to the National Survey on Drug Use and Health in the United States, fentanyl use appears to be on the rise although the most commonly misused prescription opioids are hydrocodone, oxycodone, codeine, and tramadol (UN World Drug Report, 2018). Fentanyl and fentanyl analogs are full agonists at the MOR with varying degrees of potencies, where acetylfentanyl is 5–15 times more potent than heroin (Yonemitsu et al., 2016), butyfentanyl is 7 times more potent than morphine (Steuer et al., 2017) and ocfentanil is almost 90 times more potent than morphine (Fletcher et al., 1991). There is a high demand for opioids that are popularly derived from fentanyl, which are available at a cheaper cost compared to heroin (Marchei et al., 2018; Rothberg and Stith, 2018). Fentanyl is 50 times more potent than heroin (Rothberg and Stith, 2018), it is often found in heroin samples as a cutting agent that is meant to give heroin a much higher potency, which is more favorable to drug abusers (Marchei et al., 2018). Fentanyl also causes drowsiness, sedation, euphoria (lesser than heroin and morphine), respiratory depression, anxiety, hallucinations and have associated with withdrawal symptoms such as diarrhea, abdominal cramps, anxiety, sweating, bone pain, and shivers (Stanley, 2014; Suzuki and El-Haddad, 2017; Kuczynska et al., 2018).

Rats that undergo short-term withdrawal from fentanyl self-administration (0.0032 mg/kg/infusion followed by 24 h abstinence) were found to have a disrupted brain immune response where there was an increase of inflammatory responses in the NAc simultaneously resulting in immunosuppression in the hippocampus (Ezeomah et al., 2020). It was suggested that the changes in immune outcomes in the central nervous system contribute to the relapse in OUD, however, the authors interestingly noted that the inflammation levels did not correlate with the opioid receptor expression (Grace et al., 2015; Liang et al., 2016). Cisneros and colleagues proposed that the fentanyl-associated change in the immune response contributes to neuroimmune adaptations that might drive the development of OUD, and increase the onset and severity of neurocognitive disorders (Cisneros and Cunningham, 2021). Chronic self-administration of fentanyl in rats (2.57 μg/kg per i.v. infusion, 30 days), significantly decreased ultrasonic vocalization, suggesting an aversive response to repeated fentanyl use, thus indicating negative reinforcement (Dao et al., 2021). Cessation of fentanyl administration (1.2 mg/kg/day for 14 days) also resulted in a time-dependent elevation in brain reward thresholds and somatic withdrawal signs, displaying severe deficits in brain reward function (Brujinzeel et al., 2006). In addition, chronic administration of high dose fentanyl (0.3 mg/kg/i.p. for 28 days) reduced anxiety-like behavior in rats in the open field and elevated plus maze tests (Colasanti et al., 2011; Fujii et al., 2019), reduced muscle strength and locomotion (Fujii et al., 2019) On the contrary, during withdrawal, increase in anxiety-like behavior and hyperalgesia was noticed in mice, and neither high nor low doses of fentanyl had any negative effects on the animals’ cognition (Fujii et al., 2019). In a separate study, 25 μg/kg of fentanyl reduced the grimace scale in mice and rats inflicted with injury of the infraorbital nerve (Akintola et al., 2017). In a more recent study, extended access to self-administration of a vaporized fentanyl to rats altered their behavioral economic metrics consistent with the development of an addiction-like state (McConnell et al., 2021).

The Centre for Disease Control and Prevention reported that there were a cumulative 14000 heroin users died from an overdose in the United States (Centers for Disease Control and Prevention, 2020). In addition, there is a 97.5% increase in heroin use among non-medical users of other prescription drugs suggesting increased polydrug abuse (Jones et al., 2015). Key factors behind the polydrug use of heroin and other substances are the high cost and low availability of heroin, making drug abusers seek cheaper and more lasting highs (Siegal et al., 2003; Lankenau et al., 2012; Mateu-Gelabert et al., 2015). Symptoms of heroin withdrawal include restlessness, insomnia, diarrhea, muscle and bone pain and cold flashes, depression, and nausea, peaking around 48–72 h after the last dose and may last 5–10 days (National Highway Traffic Safety Administration, 2004). Chronic administration of heroin (5 mg/kg at 12 h intervals for 34 days) impaired spatial learning and memory along with increased expression of proapoptotic proteins, relating the cognitive detriment to neural apoptotic damage (Garcia-Fuster et al., 2003; Astals et al., 2008). Withdrawal from intravenous self-administration of heroin (5 daily sessions, limited to 25 number of infusions 0.04 mg/infusion after 7 days increased to 75 number of infusions maximum), results in motivational deficit shown by a significant increase in latency to collect earned food, which was hypothesized as a consequence of the diminished perceived value of the food reward (Goldberg and Schuster, 1967; Harris and Aston-Jones, 2003; Dalley et al., 2005).

## Methamphetamine Use Disorder

Methamphetamine is a powerful psychostimulant that has been abused as a recreational drug instead of its intended use as a second-line treatment for attention deficit hyperactivity and obesity (Kish et al., 2001). Methamphetamine remains a significant public health concern over its abuse, especially in its crystalline form, where its use is rapidly increasing in East and South-East Asia (UN World Drug Report 2021). According to the UN World Drug Report 2021, the highest prevalence of Amphetamine Type Stimulant (ATS) abuse was reported in North America and the lowest in Africa. But the prevalence of non-medical use of pharmaceutical stimulants and methamphetamine was the highest in North America as well as South East Asia. Malaysia, however, reported 65.2% ATS use among its drug and substance abusers according to the National Anti-Drug Agency Report (National Anti-Drug Agency, 2019).

Clinical findings have associated chronic use of methamphetamine with manifestations of withdrawal symptoms including fatigue, sleep disturbance, dysphoria, agitation or psychomotor retardation, increased appetite, depression, and anxiety (American Psychiatric Association, 2013; Zhao et al., 2021). Anxiety and depression appear to be prominent and severe especially during the early withdrawal period (Zhang et al., 2015; Ren et al., 2017; Luan et al., 2018; Luan et al., 2018), where longer duration of methamphetamine use was associated with a higher odds ratio of depression, and co-occurring anxiety and psychotic symptoms (Ma et al., 2018). Whereas, symptoms such as craving and sleep disturbance were reported to persist as long as 4 weeks of post-abstinence (Zorick et al., 2010; Mancino and Gentry, 2011). Increased impulsivity also was reported among methamphetamine users during abstinence which is suggested to be a negative reinforcer to maintain drug use (Jones et al., 2016). In a study using the Iowa gambling task, methamphetamine dependence significantly affected inhibitory control and decision making, suggesting abnormal reward processing and inhibitory control (Fitzpatrick et al., 2020). Methamphetamine dependence also affects the cognitive ability of dependent users such as visual memory (Moon et al., 2007), attention/processing speed learning/memory, working memory, timed and executive function (Kalechstein et al., 2003), and decision making (Mizoguchi and Yamada, 2019). Moreover, it was also reported that a month of abstinence did not improve the impaired cognition of methamphetamine-dependent subjects (Simon et al., 2010).

Studies employing mice, induced methamphetamine withdrawal through various dosage regimens, where some researchers achieved this by administering the substance through varied durations such as 8 weeks (5 mg/kg, i.p, once a day, 5 days per week; Ru et al., 2019), 2 weeks (2 mg/kg, 12-h intervals; Hosseini et al., 2021), and 10 days (5 mg/kg, i.p, once a day; Georgiou et al., 2016; Jacobskind et al., 2019). Whereas, some tested escalating dose regimen for 10 days (D1: 2 mg/kg, D2: 4 mg/kg, D3-10: 6 mg/kg). Researchers using rats opted for 10 days of methamphetamine exposure (2 mg/kg, intramuscular; Li et al., 2021), some for 14 days (2 mg/kg, 12 h interval; Damghani et al., 2016), 21 days (10 mg/kg; Yasuj et al., 2019), 14 days (inhalation of methamphetamine, 1W: 5 mg/kg, 2W: 10 mg/kg; Rezaeian et al., 2020), 7 days (2 mg/kg once per day, i.p; Etaee et al., 2019), and 4 days (2.5, 5 or 7.5 mg/kg every 3 h, 3 times per day, i.p; García-Cabrerizo and García-Fuster, 2019). Withdrawal from chronic methamphetamine (various doses of methamphetamine given for 8 weeks, 21, 14, and 4 days) resulted in changes in behaviors such as anxiety and depressive symptoms when tested in the open field, sucrose preference test, forced swim test, and splash test (Damghani et al., 2016; Shabani et al., 2018; Ru et al., 2019; Yasuj et al., 2019; Rezaeian et al., 2020; Hosseini et al., 2021). In addition to this, some reported no changes in the locomotion of animals during the withdrawal period (Hosseini et al., 2021; mice; 2 mg/kg, 12-h intervals), whereas some reported increase in locomotion in methamphetamine treated animals (Rezaeian et al., 2020; rats; inhalation of methamphetamine, 1W: 5 mg/kg, 2W: 10 mg/kg). These discrepancies could be due to the differences in strains of animals tested, mode of methamphetamine intake, dose, and duration of intake as well.

## Co-Abuse of Opioid and Methamphetamine

Polysubstance use is a serious public health concern across the globe (Morley KC. et al., 2017; Lyons et al., 2019; Zuckermann et al., 2019), especially among young adults and adolescents (Tomczyk et al., 2016; Silveira et al., 2019; Willis et al., 2019; Zuckermann et al., 2019). Among the adolescents, the common polysubstance use comprised cigarettes/E-cigarettes/tobacco, alcohol, and marijuana (Tomczyk et al., 2016; Zuckermann et al., 2019, 2020; Tan et al., 2020). Systematic analysis of data from the US, United Kingdom, France, Germany, and Australia associated benzodiazepine with four-fold greater odds of misuse and six-fold greater odds of abuse with prescription opioid analgesics (Morley KI. et al., 2017). Data from the National Survey on Drug Use and Health (2015–2018; 18–64 years old) revealed that the prevalence of opioid and methamphetamine use was higher among those from the age group 18–49 (Shearer et al., 2020). In the US, methamphetamine use was significantly increased among treatment-seeking opioid users, from 18.8% in 2011 to 34.2% in 2017 (Ellis et al., 2018). In line with this, an increase in methamphetamine use was reported among primary treatment admissions, from 1 in 50 in 2008 to 1 in 8 in 2017 (Jones et al., 2020). In Malaysia, a steep increase in polydrug abuse was reported, with 8,841 polydrug abusers in 2018, by 2019 it has increased to 15,166 polydrug abusers which is around a 71.5% increase in a year’s time (National Anti-Drugs Agency, 2019). Moreover, in the US, almost half of the psychostimulant use-related deaths involve opioids, and likewise, opioid use related-deaths involve methamphetamine co-use (Ihongbe and Masho, 2016; Lancet, 2018; Gladden et al., 2019; Kariisa et al., 2019; Compton et al., 2021).

Polydrug abuse refers to the fairly common activity where drug users combine the desired effects of multiple different drugs in one administration or on separate occasions. The combination that is most popular among polydrug users is the co-use of stimulants and opioids, which is known as “speedball: combination of opioids and cocaine” (Trujillo et al., 2011) or “goofball: combination of opioids and methamphetamine” (Glick et al., 2021). Individuals with OUD often co-use methamphetamine through separate use or co-injection (Al-Tayyib et al., 2017), to balance the two drugs’ relative effects, attaining a synergistic high or mitigate the risk of overdose or withdrawal (Ellis et al., 2018; Palmer et al., 2020; Baker et al., 2021). Patients taking medications for OUD, use methamphetamine to attain an alternative high to opioids and/mitigate the sedative effects of the medications (McNeil et al., 2020; Palmer et al., 2020). Polydrug abuse also refers to the sequential use of drugs, which is the consumption of a substance after the peak effect of another substance, reportedly to alleviate withdrawal symptoms or to prolong a state of euphoria (Preston et al., 2016). Combinations most popularly included stimulant and depressant substances (Rigg and Ibañez, 2010; Silva et al., 2013) with the main motivation behind this sequential combination being the alleviation of withdrawal symptoms. However, the sequential polydrug combination does not exclude substances of the same class which aim to ease the effects of the drug (Lankenau et al., 2012; Kecojevic et al., 2015). Prolonging a high also was a motivation behind the sequential use of stimulants and opioids, which manages the opposing psychotropic effects (Valente et al., 2020). Moreover, it was reported that methamphetamine users with a history of polysubstance use (such as heroin, ketamine, and ecstasy) are more prone to develop anxiety symptoms during the early period of abstinence (Su et al., 2017). In line with this, polydrug use was associated with anxiety and depression by a 10-years prospective study (Burdzovic Andreas et al., 2015). A study involving psychostimulant-dependent patients with a history of polydrug use revealed that the severity of negative symptoms in psychostimulant-associated psychosis is not related to the psychostimulant use, but rather due to the use of opioids (Willi et al., 2016). Furthermore, it was also reported that co-use of methamphetamine and morphine results in differential physical symptoms compared to the use of morphine or methamphetamine alone. For instance, co-used patients reported increased catecholaminergic hyperstimulation of respiratory, cardiovascular, and peripheral nervous systems, and more severe neuropsychiatric symptomatology (Liu et al., 2015).

Although there have been a number of preclinical studies that attempt to characterize the polydrug abuse phenomenon, there is still insufficient evidence to completely understand the behavioral and neurochemical consequences that come with it. Chronic administration of morphine and methamphetamine increased the incidence of jumping behavior (Kaka et al., 2014), where morphine assigned rats were given cumulative doses of 5, 10, 20, 30, and 40 mg/kg per day within 5 days while methamphetamine was assigned rats were given cumulative doses of 1, 2, 4, 6 and 8 mg/kg per day for 5 days, and lastly, on day 6, a combination of 8 mg/kg methamphetamine and 40 mg/kg morphine was injected. It is indicative of an attempt to escape the test chamber due to withdrawal-induced anxiety and stress (Liu et al., 1999). Manifestation of withdrawal symptoms in the methamphetamine or morphine alone administered animals were dissimilar to the animals exposed to both methamphetamine and morphine. None of the methamphetamine-administered animals displayed escape behaviors and other behaviors such as ptosis and chewing were more pronounced in the morphine-treated animals (Kaka et al., 2014). The co-use of drugs often masks the unwanted effects of the other drug. In line with this, it was reported that the co-use of morphine and low dose methamphetamine (7.5 mg/kg and 1.0 mg/kg respectively) caused sensitization of the opioid receptor system with the psychostimulant masking the sedative effects of morphine (Ridzwan et al., 2018). The effects of methamphetamine and morphine co-use depend on the drugs’ dose and behaviors assessed, and very often synergistic effects of the drugs have been reported with concurrent co-use. An acute combination of morphine (5 mg/kg) and methamphetamine (1 mg/kg) injected subcutaneously resulted in more than twice of ambulation and more than 50% of rearing than the animals administered with each drug alone, indicating synergistic effects from the co-use (Trujillo et al., 2011). Furthermore, it was also reported that methamphetamine (0.032 mg/kg/infusion) had no reinforcing effects on rats withdrawn from morphine or morphine-dependent rats, whereas fentanyl produced high reinforcing effects on morphine withdrawn animals, and reduced effects on morphine-dependent animals. These results suggest that the reinforcing effects of methamphetamine are independent of the withdrawal or dependence state of opioid use (Seaman et al., 2021). Likewise, morphine (0.75 mg/kg i.p.) also produced synergistic effects on methamphetamine-induced (5 mg/kg i.p.) conditioned place preference and sensitization of stereotyped behaviors along with methamphetamine (Lan et al., 2009).

## Neurological Changes in Drug Dependent Brains

Addiction experts at the World Health Organization proposed in 1950 that drug addiction is primarily characterized by psychological dependence, regardless of the type of drug (Eddy and Isbell, 1959). Due to this, early psychological hypotheses linked addiction to symptoms like psychic tolerance (which was thought to be the source of increasing drug consumption) and abstinence agony (also known as withdrawal syndrome) (the presumed main obstacle to abstinence) (Solomon and Corbid, 1973). For many years, researchers speculated that the mesotelencephalic dopamine system was responsible for the rewarding effects of both opiates (such as heroin and morphine) and psychostimulants, building on the discovery that electrical stimulation of certain brain areas may produce reward (for example, cocaine, amphetamine, and methamphetamine) (Wise, 1978; Di Chiara and Imperato, 1988). Motivational effects of drug-associated signals and psychomotor sensitization to addictive substances were both linked to this system (Stewart et al., 1984). Using these neuropharmacological findings, the 1987 psychomotor stimulant theory of addiction and later theories highlighted shared psychobiological foundations for addiction, spanning drug classes, were based on these neuropharmacological breakthroughs (Wise and Bozarth, 1987; Badiani et al., 2011).

### Opioid Dependence

Acute administration of morphine to healthy volunteers (not on any type of opioids) results in positive signal changes in reward-associated regions, including the amygdala, nucleus accumbens, hippocampus, and orbitofrontal cortex (Becerra et al., 2006). Similarly, acute opioid withdrawal (naloxone-precipitated) in healthy male subjects (21–34 years old) increased neural activity in rewards-prediction and reward-association regions, including the pregenual cingulate, caudate, middle orbital gyrus, orbitofrontal gyrus, and putamen. Whereas, reduced neural activity was seen in the areas involved in the sensorimotor integration, network dysregulation, and body attentional monitoring such as the bilateral precentral and postcentral gyri, posterior insula, left anterior precuneus, and bilateral temporal lobe (Chu et al., 2015). Chronic opioid-dependent patients undergoing abstinence also recorded reductions in the midbrain-thalamic grey matter connectivity (Tolomeo et al., 2016). In a separate study on opiate-dependent patients (18–59 years old; 18 males, 11 females), baseline drug use severity and opioid withdrawal symptoms were positively correlated with the neural response to drug cues in the orbitofrontal cortex, nucleus accumbens, and amygdala. Craving, however, did not mediate such changes (Shi et al., 2021). The neurological and behavioral changes seen in opioid abstinent patients are time-dependent as well. For instance, recently withdrawn opioid-dependent patients showed reduced hedonic response to natural rewards, increased drug-related cues, increased cortisol levels compared to opioid-dependent patients that have been abstinent for 2–3 months. Furthermore, the recently withdrawn patients also had stronger dorsolateral prefrontal cortex responses to drug cues and higher cortisol levels (Bunce et al., 2015), indicating neuroplasticity in reward- and stress-associated brain regions over the abstinence period.

Heroin is greatly implicated with impulsive and poor decision-making due to its deteriorating effects in regions associated with cognitive functions (Kirby and Petry, 2004; Pirastu et al., 2006). Past fMRI findings indicate that heroin-dependent individual (HDI) groups had significant functional changes in the left prefrontal cortex, bilateral orbital frontal cortices, and left anterior cingulate gyrus as compared with control groups, where the HDI groups exhibited a disruption in the white matter structural networks (Zhang et al., 2016). Chronic heroin use is associated with white matter structural connectivity impairment in bilateral frontal lobe sub-gyrus, cingulate gyrus, medial frontal gyrus, posterior thalamic radiation, left temporal lobe sub-gyrus, and right superior frontal gyrus (Li et al., 2011) resulting in different activation patterns in the networks of reward, motivation, memory/learning and control that are heavily involved in drug abuse and addiction (Zhang et al., 2011). Functional connectivity is also compromised in chronic heroin users due to the dysregulation of brain regions (prefrontal cortex, anterior cingulate cortex, supplementary motor area, ventral striatum, insula, amygdala, and hippocampus) that lead to the decrease of the monitoring function, impairing inhibitory control and inducing deficits in stress regulation (Liu et al., 2009). The aforementioned neurological changes weakened the executive control, which manifests as increased impulsivity, based on findings from the Iowa Gambling Task (IGT) and the Barrett Impulsiveness Scale (BIS), where a positive correlation was found between poor performance in the IGT (indicating impaired decision making) with heroin use (Qiu et al., 2011; Ma et al., 2015). As for the BIS, studies investigating impulsivity in heroin-dependent individuals showed that weakened executive control is positively correlated with the BIS score (Qiu et al., 2013; Wang et al., 2016).

Acute fentanyl treatment (50 ug/kg/i.p) to rats decreased [123I]b-CIT binding to dopamine transporter in the striatum by 30%. Similarly, in a human subject, reduced [123I] b-CIT binding was noticed in the basal ganglia by 37% in the presence of fentanyl. Whereas, subacute (10 ug/kg, twice a day, i.p) in animals and following 2 weeks of drug-free period (human) recorded no significant alterations in the dopamine transporter activity. The findings indicate the differential effects of fentanyl on the reuptake of dopamine sensitive to the time frame of administrations as well (Bergström et al., 1998). In a more recent study, in nonhuman primates, intravenous self-administration of fentanyl (1 ug/kg) recorded reduced functional connectivity in the brain regions associated with the effects of opioid agonists such as the striatum, cingulate cortex, and midbrain, indicating a reduced function in motoric, cognition, and sensory-related faculties. Whereas, functional connectivity of nucleus accumbens with other regions were increased, suggesting escalation in the activities of regions associated with reward processing, drawing similarity with other types of opioid that promote addiction (Withey et al., 2022). Furthermore, chronic intake of fentanyl also has caused cognitive detriments such as opioid-related acute amnestic syndrome with MRI findings of a patient revealing restricted diffusion of the hippocampi, and 10% loss of volume in the cornu amnomis, subiculum hippocampal subfields, and dentate regions (Butler et al., 2019).

### Methamphetamine Dependence

One of the most frequently methamphetamine-associated changes in the brain is cognitive deterioration, which was shown to affect brain regions such as the prefrontal cortex and anterior cingulate cortex that involved in cognitive control, and prefrontal cortex, anterior cingulate cortex, and striatum in decision making (Sabrini et al., 2019). Chronic methamphetamine intake also caused severe gray matter deficits in the limbic, cingulate, and paralimbic cortex, reduced the hippocampal size, and the neurological findings correlated with cognitive impairment (Thompson et al., 2004). Some researchers reported significant improvement in cognitive function after withdrawal from methamphetamine use over 6 months (Proebstl et al., 2019), especially abstinence as long as 1 year was shown to normalize the cognitive function (Ludicello et al., 2010). Some reported slight improvement just after 1 month of abstinence (not significant) (Simon et al., 2010), whereas some findings indicate that even with an average abstinence period of 46 days, both abstinent and dependent patients still perform worse than the control group in cognitive assessments (Farhadian et al., 2017), suggesting a longer duration of abstinence needed to reverse the chronic methamphetamine-induced cognitive deficits. In line with this, a separate study reported that prolonged abstinence from methamphetamine use improved the grey matter volume of cognition-associated regions (Zhang et al., 2018). Such findings also imply the important roles of these brain regions in the development of methamphetamine dependence (London et al., 2015). Compared to adults, adolescent brains are more vulnerable to methamphetamine-induced alterations, even with a shorter duration of use and smaller doses, particularly affecting the frontostriatal system (Lyoo et al., 2015), which is also been reported in adults (London et al., 2015). Using an animal model, it was reported that withdrawal from chronic psychostimulant use remodels the functional architecture of the brain, causing a shift from cortical (sensory/motor) regions to the more subcortical network (Kimbrough et al., 2021). Another common symptom associated with methamphetamine dependence, that is psychosis was reported due to decreased activity in the left precentral gyrus and the left inferior frontal gyrus, and increased activity in the putamen and pallidum (Vuletic et al., 2018).

## Neurochemical Changes in Drug Dependent Brains

### Dopamine

The dopaminergic neurotransmitter system innervates brain regions associated with addiction, including the striatum, hippocampus, prefrontal cortex, amygdala, and others (Ogawa and Watabe-Uchida, 2017; Menegas et al., 2018). Variations in the inhibitory and excitatory outputs from D1 and D2 receptors of the dopamine system (Kravitz et al., 2012) somehow produces differential responses to rewards, aversive stimuli, and prediction of rewards and punishment (Ljungberg et al., 1992; Mileykovskiy & Morales 2011). D1 receptors are relatively denser in the striatum, nucleus accumbens, olfactory bulb, amygdala, hippocampus, substantia nigra, hypothalamus, and frontal cortex, while D2 receptor and its subtypes are expressed mainly in the cortex, substantia nigra, and hypothalamus (Mishra, et al., 2018).

Acute intake of opioids by opioid naïve subjects was shown to increase the dopamine release in the striatum in preclinical (Spanagel et al., 1992) and clinical (Spagnolo et al., 2019) studies, mediating the reinforcing effects of the drug. In contrast, prolonged exposure to opioids dampens the striatal dopamine release (Jia et al., 2005; Shi et al., 2008; Yeh et al., 2012) due to drug-induced adaptations in the dopamine neurotransmitter system. Such hypodopaminergic state was associated with reward deficiency syndrome, which behaviorally manifests as insufficiency in the feeling of satisfaction (Blum et al., 2015). Nevertheless, there have been conflicting findings in the dopamine levels of chronic opioid users, consistent with a human postmortem study that reported no difference in striatal dopamine transporters between opioid users and healthy deceased subjects (Kish et al., 2001; Cosgrove, 2010). However, reduced dopamine levels may induce feedback mechanisms to increase dopamine receptor expressions, which has been reported in a postmortem study of opioid users, where both D1 and D2 receptors were upregulated in the ventral tegmental area, nucleus accumbens and the amygdala (Sadat-Shirazi et al., 2018).

Psychostimulants invoke higher dopamine release in the ventral striatum compared to opioids, upon acute intake (Martinez et al., 2003; Spagnolo et al., 2019), which could be due to the direct actions of the stimulants on the dopamine transporters (Tsukada et al., 1999). Studies indicate that there is a general downregulation of dopamine receptors with stimulant use, where the action of methamphetamine is dose-dependent with methamphetamine acting primarily as a dopamine transporter blocker at low concentrations and reversing dopamine transport at high concentrations (Calipari et al., 2013; Ashok et al., 2017). Such changes cause deficits in functions of the dopamine receptors-enriched brain areas, which could be the reason why drug-dependent users crave or even relapse because the endogenous dopamine is no longer sufficient for stimulation (Wang et al., 2012; Härtel -Petri et al., 2017). The reductions in both pre-and postsynaptic dopamine receptors possibly due to the loss of dopamine neurons or damage to the dopaminergic terminals, mediated by methamphetamine-induced apoptosis through activation of caspases and formation of free radicals (De Vito and Wagner, 1989; Tata and Yamamoto, 2007; Cunha-Oliveira et al., 2008). Chronic administration of methamphetamine reduces the levels of dopamine transporters in the striatum, orbitofrontal and dorsolateral prefrontal cortex, and amygdala (McCann et al., 1998; Sekine et al., 2001; Volkow et al., 2001; Sekine et al., 2003). Animals that self-administered methamphetamine exhibited dose-dependent decreases in striatal dopamine and striatal dopamine transporter levels, as well as significant reductions in dopamine and dopamine transporter levels in the cortex (Krasnova et al., 2010). Exposure to methamphetamine reduced the levels of dopamine transporter availability which is suggested to be the mechanism behind deficits in inhibitory control that emerge in dependent individuals (Groman et al., 2012). However, there have been studies that reported no effects of methamphetamine treatment in the striatum and nucleus accumbens (Melega et al., 2008).

### Opioid Receptors

Opioid receptor subtypes are mu (MORs), kappa (KORs), and delta (DORs) (For review on opioids alone, kindly refer to Darcq and Keiffer, 2018). The MORs mediate behavioral changes such as motivational aspects (Laurent et al., 2015), impulsivity (Olmstead et al., 2009), aversion processing (Boulos, 2016), and despair-like behavior (Lutz et al., 2014). The MORs bind readily to endorphins and are mainly found in the mesocorticolimbic networks (Le Merrer et al., 2009). The KORs bind to dynorphins and act as an “anti-reward” system (Koob and Le Moal, 2008; Koob et al., 2014), mediating negative affective states such as depression, stress, dysphoria, and aversion (Crowley and Kash, 2015), that are more pronounced during the abstinence period of opioid dependence (Chavkin and Koob, 2016). The KORs are present in the striatum, hypothalamus, and periaqueductal gray (Wang, 2019). The MORs potentiates dopamine release in the nucleus accumbens, whereas the KORs inhibit dopamine release terminals in the nucleus accumbens and prefrontal cortex, hence causing dysphoria (Spanagel et al., 1992; Bals-Kubik et al., 1993). The initial positive, and negative reinforcing effects in the later stage of addiction allow the transition from recreational drug use to dependence (Gerrits et al., 2003). In rats, exposure to morphine significantly elevated the levels of accumbal MORs, but decreased levels in the ventral tegmental area (Vassoler et al., 2016). Withdrawal from chronic morphine, however, enhanced the MOR activity in the ventral tegmental area suggesting it may be an adaptive response to the elevation of cAMP levels during morphine withdrawal (Meye et al., 2012). Furthermore, withdrawal from morphine also increased MOR mRNA levels in the other reward-associated regions including the lateral hypothalamus, nucleus accumbens core, and caudate-putamen (Zhou et al., 2006). Nevertheless, the chronic opioid or withdrawal-induced mRNA changes have been inconsistent where some reported a decrease (Duttaroy & Yoburn, 2000), an increase (Sehba et al., 1997) while another reported no changes (Castelli et al., 1997), which could be due to the differences in the brain regions examined, exposure time, dose and route of opioid agonist administration.

Prolonged administration of opioids also decreased endogenous endorphin production, where administration of Fentanyl slowed down the endorphin production in patients under general anesthesia (Ballantyne, 2017). Furthermore, there is also downregulation of MORs along with the uncoupling of MORs from their ligand-gated voltage channels (Sprouse-Blum et al., 2010), causing the users to be dependent on the exogenous opioids to replicate the endogenous opioids that are unresponsive, mediating the risk for drug tolerance and addiction (Toubia and Khalife, 2019). Contrary to MORs and KORs, the DORs are not associated with the drug reward system, but more towards learning and memory (Klenowski et al., 2015; Pellissier et al., 2016), and also attenuates negative mood (Lutz et al., 2014). The DORs bind to enkephalin and are expressed in the basal ganglia (Wang, 2019), mood, motivation, and learning-related regions (Erbs et al., 2015). Chronic intake of morphine decreased the density of DOR-expressing neurons in the mice hippocampus, which persisted even after 4 weeks of abstinence (Erbs et al., 2015). This contributes to reduced inhibition of the firing activity of the hippocampus, resulting in disturbances in memory processes (Erbs et al., 2015), which is commonly reported as cognitive deficits in opioid-dependent patients, especially during the early period of abstinence (Rapeli et al., 2006).

Neuroadaptation occurs with the persistent increase in striatal MOR following methamphetamine treatment, which occurred concurrently with the emergence of anxiety-related symptoms during withdrawal (Georgiou et al., 2016). Opioid receptors do not respond similarly to methamphetamine, where a study using a 7-days regimen revealed that binding of MOR was not changed on day 2 and 5 but downregulated on day 8 then gradually returned to normal on day 11—while there were no changes in KORs and sigma opioid receptors on any given day examined (Chiu et al., 2006). Activation of the dopamine receptor is required for the increased expression of MOR mRNA, at least in the nucleus accumbens (Azaryan et al., 1996), in the presence of stimulants such as cocaine, indicating a substantial interaction between the dopaminergic and opioid system mediating the rewarding effects of stimulants. Moreover, it was also reported that the MOR is important in modulating the development of methamphetamine-induced behavioral sensitization through the dopaminergic neurotransmission (Tien and Ho, 2011). Further corroborating this were findings by Park and colleagues, who reported a decrease in the dopamine 1 receptor-ligand binding in the striatum of methamphetamine-treated MOR knockout mice (Park et al., 2011).

In a tail withdrawal test, methamphetamine (5 mg/kg) was as analgesic as 10 mg/kg morphine. However, the lower doses of methamphetamine (1 and 2 mg/kg) were not. The analgesic effects of methamphetamine were reversed by administration of naltrexone (1 mg/kg; non-selective opioid receptor antagonist), indicating the interaction between MORs and methamphetamine at higher doses. The analgesic effects of 5 mg/kg of methamphetamine were equipotent to the morphine (10 mg/kg) (Ridzwan et al., 2018). It was previously reported that daily intake of methamphetamine (2.5 mg/kg) significantly reduced the expression of MORs (Chiu et al., 2006). The researchers reported a profound decrease in the expression of MORs on day 8, not day 2 or 5, whereas Ridzwan et al. (2018) conducted the tail withdrawal test minutes upon drug administrations. At present, it is unclear whether methamphetamine able to reduce the threshold for analgesic activity by downregulating the MORs within a shorter time frame.

### Polydrug Use

Co-use of methamphetamine and morphine may alter the brain and behavior differently compared to the use of either drug alone. Previous studies have reported greater rewarding effects from the co-use of morphine and methamphetamine compared to the individual doses of the drugs (Negus et al., 1998; Ranaldi and Wise, 2000). A combined administration of methamphetamine (0.75 mg/kg) and morphine (5 mg/kg) produced higher conditioned place preference (CPP) and slower decline of CPP than equivalent individual doses of the drugs (Lan et al., 2009). Such drug-induced reinstatement has also been reported in other preclinical studies testing low doses of morphine (2 mg/kg) and methamphetamine (0.5 mg/kg) (Manzanedo et al., 2005; Tatsuta et al., 2007), which coincides with human findings where low doses of morphine and methamphetamine were reported to mediate the reinforcing effects (Lamb et al., 1991; Melega et al., 2007). Similarly, repeated administration of combined low doses of methamphetamine (0.75 and 2.5 mg/kg/day) and morphine (5 mg/kg/day) for 5 days was reported to elevate dopamine level in the nucleus accumbens compared to either drug alone (Zhu et al., 2015), indicating the higher reinforcing effects of the drugs when taken together.

Challenge administration of morphine (5 mg/kg) and methamphetamine (0.75 mg/kg) on day 40 (post chronic drugs administration) significantly increased striatal dopamine levels compared to either drug alone, but decreased dopamine turnover in the striatum (Lan et al., 2009). Whereas, challenge administration of methamphetamine alone (0.75 mg/kg) significantly decreased dopamine turnover, but morphine (5 mg/kg) produced no profound changes. The reduction in combined drug-induced dopamine turnover was lesser than methamphetamine-induced (Lan et al., 2009), indicating differential effects of the combination of drugs than either drug alone on striatal dopaminergic neurotransmission in the development of behavioral sensitization. Similar findings were also reported in a previous study, however on individual doses of methamphetamine (2 mg/kg) and morphine (10 mg/kg), where methamphetamine significantly increased dopamine release and reduced dopamine turnover in the striatum. Whereas, morphine slightly increased the dopamine levels in the striatum, and had no effects on dopamine turnover. Furthermore, the effects of methamphetamine on dopamine release and turnover were greater in the striatum than nucleus accumbens, whereas for morphine, a significant increase in the release and turnover of dopamine was seen in the nucleus accumbens than striatum (Mori et al., 2016). The findings indicate the differences in the effects of psychostimulants and opioids in the mesolimbic and nigrostriatal dopamine systems.

Effects of low doses of cocaine are enhanced in an additive manner by the addition of low dose heroin, where the drug combination significantly increased the extracellular levels of nucleus accumbens dopamine (Smith et al., 2006). The author suggests that the neurochemical effects are likely through MORs and DORs in the nucleus accumbens. However, a study showed that only the MORs in the nucleus accumbens is involved in the reinforcing effects of combined administration of heroin and cocaine where the author suggested that the DOR had no effect on speedball self-administrations because doses might have been too low and DORs is regionally specific to the shell area of the nucleus accumbens (Cornish et al., 2005). Dopamine receptors such as the D1 and D2 receptors play different roles in the combined administration of heroin and cocaine, particularly D1 receptors enhance the individual self-administration of heroin or cocaine, whereas stimulation of D2 receptors inhibits the reinforcing effects of heroin when administered together (Rowlett et al., 2007).

## Pharmacotherapy for Opioid Use Disorder and Methamphetamine Use Disorder

### Opioid Use Disorder

Methadone doses that are considered low, intermediate, and high are <50, 50–100, and >100 mg/day, respectively whereas the dose for methadone maintenance treatment varies between 30 and 125 mg/day (Robles et al., 2002). Oral intake of methadone for over 3 months improved the quality of life and reduced transmission of blood-borne diseases among opioid-dependent patients (Ali et al., 2018). Despite the efficacy of MMT in harm reduction, there are still other clinical concerns regarding the safety of the therapy. The patients undergoing MMT often have co-morbidities, where they are already on prescription drugs, therefore when added with methadone it might lead to unwanted drug-drug interactions, such as the development of “opioid withdrawal-like symptoms” in the case of efavirenz and zidovudine which are common treatments for HIV patients who are highly prevalent under the MMT program (George et al., 2018). Patients with opioid dependence also tend to have higher rates of mood disorders and other illicit substance abuse, where a combination of these factors may lead to the possibility of central nervous system effects and worsening behavioral symptoms (George et al., 2018). Other potential adverse effects of methadone include nephrotoxicity (Atici et al., 2005; Lentine et al., 2015) and cardiotoxicity (Kumar, 2010).

Buprenorphine is an opioid partial agonist that sustains abstinence, delays the time of resumption to opioid use, and retains patients in treatment (Schottenfeld et al., 2005). Buprenorphine has a long half-life of 24–60 h and typical dosages for maintenance treatment are 8–16 mg/day (Walsh et al., 1994; Kampman and Jarvis, 2015). Due to problems associated with diversion and abuse with buprenorphine treatment, the buprenorphine/naloxone combination tablet was introduced (Vicknasingam et al., 2010). Other combinations of buprenorphine exist as well; however, they were injected illicitly, which instead increased opioid dependence (Yokell et al., 2011).

Methamphetamine use was reported among treatment-seeking OUD patients with a prevalence of 85% in the United States (Ellis et al., 2018), where most of these patients recorded a significantly higher percentage of positive results in the urine morphine test which indicated relapse of opioid use (Liu et al., 2018). In addition, methamphetamine use was also associated with a higher risk of buprenorphine non-retention (Tsui et al., 2020). Whereas, another study found no such associations between methamphetamine use and opioid abstinence in OUD pharmacological management (methadone) (Smyth et al., 2018). The overall impact of methamphetamine use on OUD treatment outcomes are still unclear, but patients have described a balancing effect of the drugs (increases functionality of the drug that is associated with a lower perceived need for medications for OUD) that lead towards non-retention of treatment (Mcneil et al., 2020) (Table 1).

**TABLE 1:** Current treatment in opioid dependence.

No	Reference	Drug name	Design of study	Number of patients	Drug administration	Dosage	Duration of study	Mechanism of action	Results	Conclusion
1	Ali et al (2018)	Methadone	Cross-sectional	1,233 (99.1% Male)	Oral	—	3 months	Opioid agonist	Improved quality of life, effective in reducing transmission of blood borne viruses	Recommended MMT to be continued
2	Crist et al. (2018)	Methadone	Randomized, open label trial	764 (68.7% Male)	Oral	Flexible dosing	24 weeks	Opioid agonist	Genotype for 5-HTTLPR in the SLC6A4 gene was nominally associated with dropout rate when the methadone and buprenorphine/naloxone groups were combined	Patients with the S/S genotype at 5-HTTLPR in SLC6A4 or the Val/Val genotype at Val158Met in COMT may require additional treatment to improve their chances of completing addiction treatment
3	Ledberg, (2017)	Methadone	—	441	Oral	—	—	Opioid agonist	Not being in treatment was associated with a significantly increased hazard of dying	Changes in regulations that minimizes the time off treatment are therefore likely to reduce the mortality rates among clients of MMT-programs
4	George et al. (2018)	Methadone	Case series	7 (6 Male, 1 Female)	Oral	—	—	Opioid agonist	In each individual case, there is a need to avoid adverse effects, drug–drug interactions and overdosing, especially in the presence of comorbidities	despite methadone being an effective therapy for opioid dependence, there is a need for other effective therapies, such as naltrexone and buprenorphine– naloxone, to be made available to physicians in both the public and private sector
5	Schwartz et al. (2017)	Methadone	Two arm open label randomized trial	300 (59% Male)	Oral	—	12 months	Opioid agonist	There were no significant differences	Patient centred treatment does not appear to be more effective than methadone treatment as usual
6	Chen et al. (2013)	Contingency management	—	126 (92.3% Male; Mean age 38.1 years)	—	—	12 weeks	—	The retention rate and negative urine testing rate were higher in the CM group compared to the usual treatment. Compared participants who received usual treatment, CM participants missed less visits and more likely to submit a negative urine sample	CM intervention significantly improved attendance and reduced drug use in China
7	Hser et al. (2011)	Contingency management	—	259 (76.2% Male; Mean age 38; All reported drug use 30 days prior)	—	—	12 weeks	—	Relative to treatment as usual, better retention was observed among the incentive group, as well as submission of negative urine samples and longer duration of sustained abstinence	Contingency management improves treatment retention and drug abstinence in methadone maintenance treatment clinics in China
8	Chawarski et al. (2011)	Behavioral drug and HIV risk reduction counseling	—	37 (81% Male; Mean age 36.7 years)	—	—	3 months	—	Participants achieved greater reductions in HIV risk behaviors and illicit opiate use	A promising approach to improve the efficacy of standard MMT services in China

A retrospective study in Rhode Island was the first to investigate the efficacy of MMT in fentanyl abuse, which reported that the majority of patients that underwent 6 months of methadone maintenance achieved abstinence (89%), but the relapse rate was still high (59%) (Stone et al., 2018). Silverstein and colleagues analyzed qualitative data from 63 interviews, to investigate the presence of illicit non-pharmaceutical fentanyl in the current environment and how it has affected practices of non-prescribed use of buprenorphine. The participants consisted of OUD patients on non-prescribed buprenorphine, where they used illicit opioids such as buprenorphine not in seek of euphoria, instead as a form of self-treatment. However, some reported that non-pharmaceutical fentanyl defeated the harm reduction brought by buprenorphine as there were unanticipated experiences of withdrawals (Silverstein et al., 2019). However, the Zurich or Bernese method has been considered a valuable modification to buprenorphine induction for the treatment of fentanyl abuse where it utilizes micro-dosing of buprenorphine. Micro-dosing or micro-induction of buprenorphine is a method of administering buprenorphine in small incremental doses during initiation of treatment that slowly builds buprenorphine at opioid receptors without precipitating withdrawal (Ahmed et al., 2021). Overlapping induction of buprenorphine while being on full mu agonists such as methadone is feasible, where patients experienced very mild opioid withdrawal and craving (Hämmig et al., 2016).

Psychosocial interventions in conjunction with medications for the treatment of opioid addiction are approved as a part of comprehensive treatment for opioid addiction such as contingency management (CM) and cognitive behavioral therapy (CBT), with the majority focusing on methadone treatment (Dugosh et al., 2016). Studies showed that CM participants attended more days of treatment and had longer durations of continued abstinence (Hser et al., 2011; Chen et al., 2013) while CBT participants displayed significant improvements in their positive appraisal at the 6-months assessment and lower emotional discharge at the 12-months assessment compared to control group MMT alone (Kouimtsidis et al., 2012). Other psychosocial interventions include behavioral drug and HIV risk-reduction counseling, motivational interviewing, acceptance and commitment therapy, general supportive counseling, and web-based behavioral interventions (Chawarski et al., 2011; Stotts et al., 2012; Gu et al., 2013; Marsch et al., 2014).

### Methamphetamine Use Disorder

In addition to OUD, MMT is also prescribed to chronic methamphetamine users as treatment (Singh et al., 2020). Comparison between the methadone (a full agonist of the MORs) and buprenorphine (a partial agonist of the MORs) in the reduction of methamphetamine craving revealed more significant craving-attenuating effects of buprenorphine during methamphetamine withdrawal (Ahmadi and Razeghian Jahromi, 2017). In another study, buprenorphine significantly reduced methamphetamine cravings compared to bupropion (weak inhibitor of dopamine and norepinephrine reuptake) for 14 days (Ahmadi et al., 2019). Bupropion also did not significantly increase abstinence duration in methamphetamine-dependent patients compared to placebo (Anderson et al., 2015).

N-acetyl cysteine (NAC) reduces the synaptic release of glutamate (Dean et al., 2012). Preclinical studies and early pilot clinical investigations suggested that NAC may be useful in the treatment of methamphetamine dependence, showing good efficacy in suppressing methamphetamine craving however, there was no report made on methamphetamine use outcomes (Ebrahimi et al., 2015). A combination of NAC and naltrexone was found to be no more superior than a placebo in reducing methamphetamine craving (Grant et al., 2010). Modafinil (dopamine reuptake inhibitor), was not effective in decreasing methamphetamine consumption compared to the placebo (Heinzerling et al., 2010). Whereas, another study reported that those who were compliant in taking the modafinil drug were more likely to reduce drug use (Anderson et al., 2012). Both controlled trials were comparing modafinil daily doses ranging from 200 to 400 mg.

Varenicline at 1 mg (an α4β2 nicotinic receptor partial agonist and α7 nicotinic receptor full agonist) taken twice daily for 9 weeks had no significant effects on end-of-treatment-abstinence and treatment effectiveness score compared to placebo in methamphetamine dependence (Briones et al., 2018). Sustained release of methylphenidate (daily dosing regimen of 18 mg at week 1, 36 mg at week 2, and 54 mg for the remaining weeks) on the other hand was safe and well-tolerated among active methamphetamine users and significantly reduced methamphetamine use, craving, and depressive symptoms (Tiihonen et al., 2012; Miles et al., 2013; Rezaei et al., 2015). Methylphenidate is a dopamine reuptake inhibitor (Karila et al., 2010).

Sixteen weeks of CBT reduced methamphetamine dependence and improved the psychological well-being of patients undergoing methadone therapy. The 30 participants in the treatment group became abstinent at post-test and remained abstinent at the 3-months follow-up (Shakiba et al., 2018). The CBT also reduced craving among methamphetamine abusers living with HIV/AIDS (Jalali et al., 2018). Significant reductions in methamphetamine use and psychiatric symptoms were seen following the psychosocial interventions (Polcin et al., 2014; Rawson et al., 2021). The matrix model, which is a multi-component treatment adopting elements of CBT, MI, family, and group therapy, was found to be more effective in increasing methamphetamine abstinence compared to treatment as usual (CBT only) (Rawson et al., 2021). It is reported that sessions of both MI and CBT significantly increased abstinence as well (Baker et al., 2004, 2005). The treatment combining MI and CBT was found to be effective in improving abstinence where participants reported fewer negative consequences of methamphetamine use at follow-up and intensive matrix program produced a higher abstinence rate compared to CBT alone (Smout et al., 2010) (Table 2).

**TABLE 2:** Current treatment in methamphetamine dependence.

No	Reference	Drug name	Design of study	Number of patients	Drug administration	Dosage	Duration of study	Mechanism of action	Results	Conclusion
1	Mousavi et al. (2015)	N-acetylcysteine	Double blind controlled crossover	23 (82.6% male)	Oral	1,200 mg/day	8 weeks	Reduction of gluatamate release from synapses	Craving score increased indicating that NAC has a limited enduring effect for relapse prevention	NAC showed good efficacy in suppressing METH craving, and may be a useful pharmacological treatment for METH dependency
2	Trivedi et al. (2021)	Naltrexone Bupropion	Double blind, placebo controlled trial	403 (68.7% Male; Mean age 41 years)	Oral and injectables	Naltrexone (380 mg every 3 weeks) Burpropion (450 mg per day)	12 weeks	Opioid receptor antagonist	the response over a period of 12 weeks among participants who received extended-release injectable naltrexone plus oral extended-release bupropion was low but was higher than participants who received placebo	In persons with moderate or severe methamphetamine use disorder, treatment with the combination of extended-release injectable naltrexone and daily oral extended-release bupropion over a period of 12 weeks resulted in a higher response than placebo
3	Ray et al., 2015	Naltrexone	Double blind, randomized, crossover, placebo controlled trial	30 (73.3% Male; Mean age 36.9 years)	Oral	50 mg	2 weeks	Opioid receptor antagonist	NTX attenuated cue-induced craving as compared with placebo, as well as cue-provoked increases in heart rate and diastolic blood pressure	NTX is superior to placebo in attenuating cue-induced craving for MA, as well as several dimensions of MA-induced subjective effects (eg, “stimulated” and “crave drug”) measured during controlled MA administration
4	Shakiba et al. (2018)	Cognitive behavioral therapy	Randomized controlled trial	200	—	—	16 weeks	—	16 sessions of CBT led to significant reduction of methamphetamine use and improved psychological well-being	Cognitive behavioral therapy can be a good option for methamphetamine problem in methadone treatment
5	Jalali et al. (2018)	Cognitive behavioral therapy	Quasi-experimental	60 (Mean age 31.8 years)	—	—	12 weeks	—	methamphetamine craving reduced among the abusers living with HIV/AIDS.	the principle and techniques of cognitive-behavioral therapy and the benefits of group therapy have an effect on craving among methamphetamine abusers living with HIV/AIDS.
6	Polcin et al. (2014)	Motivational interviewing	—	217 (51.3% Male)	—	—	8 weeks	—	Reductions in Addiction Severity Index psychiatric severity scores and days of psychiatric problems during the past 30 days were found for clients in the intensive motivational interview group	Intensive MI may help alleviate co-occuring psychiatric problems that are unaffected by shorter MI interventions
7	Ahmadi and Razeghian Jahromi, (2017)	Buprenorphine Methadone	Double blind clinical trial	40 (100% Male)	Oral	8 mg of buprenorphine daily and 40 mg of methadone daily	17 days	—	the craving in the buprenorphine group was significantly lower than that in the methadone group starting on the 10th day. Therefore, buprenorphine was more effective than methadone	Buprenorphine is effective for decreasing methamphetamine craving during methamphetamine withdrawal and more effective than methadone
8	Anderson et al. (2012)	Modafinil	Randomized, double blind, placebo controlled	210	Oral	400 mg	16 weeks	Dopamine reuptake inhibitor	No significant difference between modafinil group and placebo	Data suggest that modafinil, plus group behavioral therapy, was not effective in decreasing methamphetamine use
9	Heinzerling et al. (2010)	Modafinil	Randomized, double blind, placebo controlled	71 (73.5% Male)	Oral	400 mg	12 weeks	Dopamine reuptake inhibitor	No statiscally significant effects for modafinil on methamphetamine use, retention, depressive symptoms, cravings	Modafinil was no more effective than placebo
10	Anderson et al. (2015)	Bupropion	Randomized double blind placebo controlled	204 (65.3% Male; Mean age 39.1 years)	Oral	150 mg	12 weeks	Weak inhibitor of norepinephrine and dopamine uptake	No significant increase in abstinence in methamphetamine dependent participants compared to placebo	bupropion did not increase abstinence in MA dependent participants
11	Ahmadi et al. (2019)	Buproprion Buprenorphine	Randomized, double blind, placebo controlled	65	Oral	300 mg bupropion/8 g of buprenorphine	2 weeks	—	Reduction of craving in the buprenorphine group was significantly more than the bupropion group	Both medications were effective in the reduction of methamphetamine cravings. Reduction of craving in the buprenorphine group was significantly more than the bupropion group
12	Briones et al. (2018)	Varenicline	Randomized, double blind, placebo controlled	52 (63% Male; Mean age 34.4 years)	Oral	starting at 0.5 mg daily for day 1–3, then 0.5 mg twice daily for day 4–7, and 1 mg twice daily from day 8 until completion of the medication phase	9 weeks	An alpha4beta2 nicotinic receptor partial agonist and alpha7 nicotinic receptor full agonist	There was no significant difference between varenicline and placebo	1 mg varenicline was not an effective treatment for methamphetamine dependence
13	Rezaei et al. (2015)	Methylphenidate	Randomized double blind placebo controlled	56	Oral	18 mg/day during first week, 36 mg/day in second week and 54 mg/day for the remaining 8 weeks	10 weeks	Dopamine reuptake inhibitor	Methylphenidate group showed less craving scores compared to the placebo group, and greater improvement in the depressive symptom scores	Sustained-released methylphenidate was safe and well tolerated among active methamphetamine users and significantly reduced methamphetamine use, craving and depressive symptoms
14	Ling et al., 2014	Methylphenidate	Randomized double blind placebo controlled	110 (81.8% Male; Mean age 38.7 years)	Oral	18 mg/day during first week, 36 mg/day in second week and 54 mg/day for the remaining 8 weeks	10 weeks	Dopamine reuptake inhibitor	The methylphenidate group had lower craving scores than the placebo group in the last 30 days	Methylphenidate may lead to a reduction in concurrent methamphetamine use when provided as treatment for patients undergoing behavioral support for moderate to severe methamphetamine use disorder
15	Miles et al. (2013)	Methylphenidate	Randomized double blind placebo controlled	79 (62.3% Male; Mean age 37.5 years)	Oral	19 mg/day during first week, 36 mg/day in second week and 54 mg/day until end of week 22	22 weeks	Dopamine reuptake inhibitor	No statistically significant difference in the percentage of positive urines between the methylphenidate and placebo arms	The trial failed to replicate earlier findings

### Polysubstance Abuse

Naltrexone subcutaneous implants (1,000 mg) for 12 weeks showed higher retention of patients with decreased use of heroin and methamphetamine, providing some of the earliest evidence for effective pharmacological treatment (Tiihonen et al., 2012). Furthermore, a combination of 0.3 mg/kg buprenorphine and 1.0 mg/kg naltrexone treatment in an 18-days experiment was reported to reduce relapse in the cocaine and morphine co-administration (McCann, 2008; Cordery et al., 2014). Apart from that, based on heroin-dependent polydrug abusers with contingency management and buprenorphine maintenance (2 mg for 5 weeks), it was suggested that for patients who have already achieved polydrug abstinence, contingency management may enhance treatment outcomes. However, participants generally did not produce any significant treatment outcomes which could possibly be due to the population sample where buprenorphine-maintained polydrug abusers continued to use illicit opiates at fairly high levels (Downey et al., 2000). The use of 0.3 mg/kg buprenorphine and 1.0 mg/kg naltrexone treatment was studied in morphine and methamphetamine polydrug dependent mice and results show that the combination successfully attenuated polydrug-reinstatement (Suhaimi, 2017).

Methadone maintenance at a relatively high dose of 30 mg/kg a day in 3 h, attenuated heroin and cocaine-seeking behavior, possibly by reducing the incentive value of drug-related cues (Leri et al., 2004). In a separate study, hypothermia was observed after 360 min of coadministration of methamphetamine and morphine. The cooling was beneficial after 30 min (golden hour) of co-administration. During this early stage, methamphetamine plus morphine-induced significant hyperthermia (Namiki et al., 2005). Another study also proved that the lethal effect induced by co-administration of methamphetamine and morphine was significantly and almost completely diminished by cooling from 30 to 90 min afterward, with normal behaviors such as grooming, sniffing, and rearing returned with the normalization of colonic temperature (Mori et al., 2007). Both studies indicated a “golden hour” of between 30 and 90 min for cooling in the treatment of subacute toxicity and lethality produced by the co-administration (Table 3).

**TABLE 3:** Current treatment in polydrug dependence.

No	Reference	Drug name	Design of study	Number of patients	Drug administration	Dosage	Duration of study	Mechanism of action	Results	Conclusion
1	Tiihonen et al. (2012)	Naltrexone	Randomized, double blind, placebo controlled trial	100	Subcutaneous	1,000 mg naltrexone	10 weeks	Opioid receptor antagonist	Relative to placebo, the naltrexone implant resulted in higher retention in the study, decreased heroine and amphetamine use and improved clinical condition of patients	Naltrexone implant acts as an effective pharmacological treatment for this type of polydrug dependence
2	Downey et al. (2000)	Contigency managemtent with buprenorphine maintenance	—	41 (Mean age 40 years old, 25 male)	—	—	12 weeks	—	Results indicated that treatment produced significant reductions in drug problems over time, but there were no significant difference among treatment conditions	Hypothesis that voucher based reinforcement therapy would promote enhanced polydrug abstinence was not supported

## Conclusion

The prevalence and risk associated with polydrug use are threats to the current public health resources worldwide. Thus, improving and pooling the understanding of mechanisms behind individual drugs and their combined use is essential to accurately reflect their effects on the neurochemical systems. However, this knowledge is still limited, especially in its polydrug combinations that can contribute to unique addiction potential and the development of addictive behaviors. It is important to appreciate novel preclinical experiments that investigate the pathophysiology and pharmacotherapy targeting the mono and polydrug abuse of morphine and methamphetamine. Given how complex addiction as a disease is with many powerful elements playing their roles, we need to understand the mechanisms behind the relationship between polydrug abuse and addiction to determine better and more effective treatments for this ongoing public health crisis.

## References

[B1] Ahmadi J. Razeghian Jahromi L. (2017). Comparing the Effect of Buprenorphine and Methadone in the Reduction of Methamphetamine Craving: a Randomized Clinical Trial. Trials 18 (1), 259–268. 10.1186/s13063-017-2007-3 28587620 PMC5461765

[B2] Ahmadi J. Sahraian A. Biuseh M. (2019). A Randomized Clinical Trial on the Effects of Bupropion and Buprenorphine on the Reduction of Methamphetamine Craving. Trials 20 (1), 468–477. 10.1186/s13063-019-3554-6 31362784 PMC6668115

[B3] Ahmed S. Bhivandkar S. Lonergan B. B. Suzuki J. (2021). Microinduction of Buprenorphine/naloxone: a Review of the Literature. Am. J. Addict. 30 (4), 305–315. 10.1111/ajad.13135 33378137

[B4] Akintola T. Raver C. Studlack P. Uddin O. Masri R. Keller A. (2017). The Grimace Scale Reliably Assesses Chronic Pain in a Rodent Model of Trigeminal Neuropathic Pain. Neurobiol. Pain 2, 13–17. 10.1016/j.ynpai.2017.10.001 29450305 PMC5808980

[B5] Al-Tayyib A. Koester S. Langegger S. Raville L. (2017). Heroin and Methamphetamine Injection: an Emerging Drug Use Pattern. Subst. Use Misuse 52 (8), 1051–1058. 10.1080/10826084.2016.1271432 28323507 PMC5642954

[B6] Ali N. Aziz S. A. Nordin S. Mi N. C. Abdullah N. Paranthaman V. (2018). Evaluation of Methadone Treatment in Malaysia: Findings from the Malaysian Methadone Treatment Outcome Study (MyTOS). Subst. Use Misuse 53 (2), 239–248. 10.1080/10826084.2017.1385630 29116878

[B7] American Psychiatric Association (2013). Substance-related and Addictive Disorders, Diagnostic and Statistical Manual of Mental Disorders: DSM-5. American Psychiatric Association.

[B8] Anderson A. L. Li S. H. Biswas K. McSherry F. Holmes T. Iturriaga E. (2012). Modafinil for the Treatment of Methamphetamine Dependence. Drug Alcohol Depend 120 (1-3), 135–141. 10.1016/j.drugalcdep.2011.07.007 21840138 PMC3227772

[B9] Anderson A. L. Li S. H. Markova D. Holmes T. H. Chiang N. Kahn R. Campbell J. Dickerson D. L. Galloway G. P. Haning W. Roache J. D. Stock C. Elkashef A. M. (2015). Bupropion for the Treatment of Methamphetamine Dependence in Non-daily Users: a Randomized, Double-Blind, Placebo-Controlled Trial. Drug Alcohol Depend 150, 170–174. 10.1016/j.drugalcdep.2015.01.036 25818061 PMC4388163

[B10] Anraku T. Ikegaya Y. Matsuki N. Nishiyama N. (2001). Withdrawal from Chronic Morphine Administration Causes Prolonged Enhancement of Immobility in Rat Forced Swimming Test. Psychopharmacology (Berl) 157 (2), 217–220. 10.1007/s002130100793 11594449

[B11] Ashok A. H. Mizuno Y. Volkow N. D. Howes O. D. (2017). Association of Stimulant Use with Dopaminergic Alterations in Users of Cocaine, Amphetamine, or Methamphetamine: a Systematic Review and Meta-Analysis. JAMA psychiatry 74 (5), 511–519. 10.1001/jamapsychiatry.2017.0135 28297025 PMC5419581

[B12] Astals M. Domingo-Salvany A. Buenaventura C. C. Tato J. Vazquez J. M. Martín-Santos R. (2008). Impact of Substance Dependence and Dual Diagnosis on the Quality of Life of Heroin Users Seeking Treatment. Subst. Use Misuse 43 (5), 612–632. 10.1080/10826080701204813 18393080

[B13] Atici S. Cinel I. Cinel L. Doruk N. Eskandari G. Oral U. (2005). Liver and Kidney Toxicity in Chronic Use of Opioids: an Experimental Long Term Treatment Model. J. Biosci. 30 (2), 245–252. 10.1007/bf02703705 15886461

[B14] Azaryan A. V. Clock B. J. Cox B. M. (1996). Mu Opioid Receptor mRNA in Nucleus Accumbens Is Elevated Following Dopamine Receptor Activation. Neurochem. Res. 21 (11), 1411–1415. 10.1007/BF02532382 8947931

[B15] Bach P. Hayashi K. Milloy M. J. Nosova E. Kerr T. Wood E. (2020). Characterising the Increasing Prevalence of crystal Methamphetamine Use in Vancouver, Canada, from 2006-2017: A Gender-Based Analysis. Drug Alcohol. Rev. 39 (7), 932–940. 10.1111/dar.13126 32666650 PMC7683370

[B16] Badiani A. Belin D. Epstein D. Calu D. Shaham Y. (2011). Opiate versus Psychostimulant Addiction: the Differences Do Matter. Nat. Rev. Neurosci. 12 (11), 685–700. 10.1038/nrn3104 21971065 PMC3721140

[B17] Baker A. Lee N. K. Claire M. Lewin T. J. Grant T. Pohlman S. (2005). Brief Cognitive Behavioural Interventions for Regular Amphetamine Users: a Step in the Right Direction. Addiction 100 (3), 367–378. 10.1111/j.1360-0443.2005.01002.x 15733250

[B18] Baker A. Lee N. K. Jenner L. (2004). Models of Intervention and Care for Psychostimulant Users. NDS Monograph Series.

[B19] Baker R. Leichtling G. Hildebran C. Pinela C. Waddell E. N. Sidlow C. (2021). "Like Yin and Yang": Perceptions of Methamphetamine Benefits and Consequences Among People Who Use Opioids in Rural Communities. J. Addict. Med. 15 (1), 34–39. 10.1097/adm.0000000000000669 32530888 PMC7734765

[B20] Ballantyne J. C. (2017). Opioids for the Treatment of Chronic Pain: Mistakes Made, Lessons Learned, and Future Directions. Anesth. Analg 125 (5), 1769–1778. 10.1213/ane.0000000000002500 29049121

[B21] Ballester J. Valentine G. Sofuoglu M. (2017). Pharmacological Treatments for Methamphetamine Addiction: Current Status and Future Directions. Expert Rev. Clin. Pharmacol. 10 (3), 305–314. 10.1080/17512433.2017.1268916 27927042

[B22] Bals-Kubik R. Ableitner A. Herz A. Shippenberg T. S. (1993). Neuroanatomical Sites Mediating the Motivational Effects of Opioids as Mapped by the Conditioned Place Preference Paradigm in Rats. J. Pharmacol. Exp. Ther. 264, 489–495. 8093731

[B23] Becerra L. Harter K. Gonzalez R. G. Borsook D. (2006). Functional Magnetic Resonance Imaging Measures of the Effects of Morphine on central Nervous System Circuitry in Opioid-Naive Healthy Volunteers. Anesth. Analg 103 (1), 208. 10.1213/01.ane.0000221457.71536.e0 16790655

[B24] Bergström K. A. Jolkkonen J. Kuikka J. T. Åkerman K. K. Viinamäki H. Airaksinen O. (1998). Fentanyl Decreases β‐CIT Binding to the Dopamine Transporter. Synapse 29 (4), 413–415. 10.1002/(SICI)1098-2396(199808)29:4<413::AID-SYN13>3.0.CO;2-R 9661259

[B25] Blum K. Thanos P. K. Oscar-Berman M. Febo M. Baron D. Badgaiyan R. D. (2015). Dopamine in the Brain: Hypothesizing Surfeit or Deficit Links to Reward and Addiction. J. Reward Defic Syndr. 1, 95–104. 10.17756/jrds.2015-016 27398406 PMC4936401

[B26] Boulos L. J. (2016). “Mu Opioid Receptors in the Habenula: Dissecting Reward and Aversion in Addiction,” in Program No. 78.11. 2016 Neuroscience Meeting Planner (San Diego, CA: Society for Neuroscience).

[B27] Briones M. Shoptaw S. Cook R. Worley M. Swanson A. N. Moody D. E. (2018). Varenicline Treatment for Methamphetamine Dependence: a Randomized, Double-Blind Phase II Clinical Trial. Drug Alcohol Depend 189, 30–36. 10.1016/j.drugalcdep.2018.04.023 29860057 PMC6391991

[B28] Buckman S. G. Hodgson S. R. Hofford R. S. Eitan S. (2009). Increased Elevated Plus Maze Open-Arm Time in Mice during Spontaneous Morphine Withdrawal. Behav. Brain Res. 197 (2), 454–456. 10.1016/j.bbr.2008.09.035 18955087

[B29] Bunce S. C. Harris J. D. Bixler E. O. Taylor M. Muelly E. Deneke E. (2015). Possible Evidence for Re-regulation of HPA axis and Brain Reward Systems over Time in Treatment in Prescription Opioid-dependent Patients. J. Addict. Med. 9 (1), 53–60. 10.1097/adm.0000000000000087 25469651

[B30] Burdzovic Andreas J. Lauritzen G. Nordfjaern T. (2015). Co-occurrence between Mental Distress and Poly-Drug Use: a Ten Year Prospective Study of Patients from Substance Abuse Treatment. Addict. Behav. 48, 71–78. 10.1016/j.addbeh.2015.05.001 26004857

[B31] Butler P. M. Barash J. A. Casaletto K. B. Cotter D. L. La Joie R. Geschwind M. D. (2019). An Opioid-Related Amnestic Syndrome with Persistent Effects on Hippocampal Structure and Function. J. Neuropsychiatry Clin. Neurosci. 31 (4), 392–396. 10.1176/appi.neuropsych.19010017 31177905 PMC7469957

[B32] Calipari E. S. Ferris M. J. Salahpour A. Caron M. G. Jones S. R. (2013). Methylphenidate Amplifies the Potency and Reinforcing Effects of Amphetamines by Increasing Dopamine Transporter Expression. Nat. Commun. 4 (1), 2720–2810. 10.1038/ncomms3720 24193139 PMC4017736

[B33] Campbell L. A. Avdoshina V. Rozzi S. Mocchetti I. (2013). CCL5 and Cytokine Expression in the Rat Brain: Differential Modulation by Chronic Morphine and Morphine Withdrawal. Brain Behav. Immun. 34, 130–140. 10.1016/j.bbi.2013.08.006 23968971 PMC3795805

[B34] Castelli M. P. Melis M. Mameli M. Fadda P. Diaz G. Gessa G. L. (1997). Chronic Morphine and Naltrexone Fail to Modify Mu-Opioid Receptor mRNA Levels in the Rat Brain. Brain Res. Mol. Brain Res. 45 (1), 149–153. 10.1016/S0169-328X(96)00305-1 9105683

[B35] Centers for Disease Control and Prevention (2020). Increase in Fatal Drug Overdoses across the United States Driven by Synthetic Opioids before and during the COVID-19 Pandemic. CDC Health Alert Network.

[B36] Chavkin C. Koob G. F. (2016). Dynorphin, Dysphoria, and Dependence: the Stress of Addiction. Neuropsychopharmacology 41 (1), 373–374. 10.1038/npp.2015.258 PMC4677142 26657953

[B37] Chawarski M. C. Zhou W. Schottenfeld R. S. (2011). Behavioral Drug and HIV Risk Reduction Counseling (BDRC) in MMT Programs in Wuhan, China: a Pilot Randomized Clinical Trial. Drug Alcohol Depend 115 (3), 237–239. 10.1016/j.drugalcdep.2010.09.024 21159452 PMC3076517

[B38] Chen W. Hong Y. Zou X. McLaughlin M. M. Xia Y. Ling L. (2013). Effectiveness of Prize-Based Contingency Management in a Methadone Maintenance Program in China. Drug Alcohol Depend 133 (1), 270–274. 10.1016/j.drugalcdep.2013.05.028 23831409

[B39] Chiu C. T. Ma T. Ho I. K. (2006). Methamphetamine-induced Behavioral Sensitization in Mice: Alterations in Mu-Opioid Receptor. J. Biomed. Sci. 13 (6), 797–811. 10.1007/s11373-006-9102-x 16847721 PMC2925105

[B40] Chu L. F. Lin J. C. Clemenson A. Encisco E. Sun J. Hoang D. (2015). Acute Opioid Withdrawal Is Associated with Increased Neural Activity in Reward-Processing Centers in Healthy Men: A Functional Magnetic Resonance Imaging Study. Drug Alcohol Depend 153, 314–322. 10.1016/j.drugalcdep.2015.04.019 26059463

[B41] Cisneros I. E. Cunningham K. A. (2021). Self-administered Fentanyl Profoundly Impacts Rat Brain Innate Immune Targets. Neuropsychopharmacology 46 (1), 247. 10.1038/s41386-020-00853-y 32908244 PMC7689450

[B42] Colasanti A. Rabiner E. A. Lingford-Hughes A. Nutt D. J. (2011). Opioids and Anxiety. J. Psychopharmacol. 25 (11), 1415–1433. 10.1177/0269881110367726 20530588

[B43] Compton W. M. Valentino R. J. Du Pont R. L. (2021). Polysubstance Use in the U.S. Opioid Crisis. Mol. Psychiatry 26, 41–50. 10.1038/s41380-020-00949-3 33188253 PMC7815508

[B44] Cordery S. F. Taverner A. Ridzwan I. E. Guy R. H. Delgado-Charro M. B. Husbands S. M. (2014). A Non-rewarding, Non-aversive Buprenorphine/naltrexone Combination Attenuates Drug-Primed Reinstatement to Cocaine and Morphine in Rats in a Conditioned Place Preference Paradigm. Addict. Biol. 19 (4), 575–586. 10.1111/adb.12020 23240906

[B45] Cornish J. L. Lontos J. M. Clemens K. J. McGregor I. S. (2005). Cocaine and Heroin ('speedball') Self-Administration: the Involvement of Nucleus Accumbens Dopamine and μ-opiate, but Not δ-opiate Receptors. Psychopharmacology 180 (1), 21–32. 10.1007/s00213-004-2135-9 15682301

[B46] Cosgrove K. P. (2010). Imaging Receptor Changes in Human Drug Abusers. Curr. Top. Behav. Neurosci. 3, 199–217. 10.1007/7854_2009_24 21161754 PMC3760378

[B47] Crist R. C. Li J. Doyle G. A. Gilbert A. Dechairo B. M. Berrettini W. H. (2018). Pharmacogenetic Analysis of Opioid Dependence Treatment Dose and Dropout Rate. Am. J. Drug Alcohol. Abuse 44 (4), 431–440. 10.1080/00952990.2017.1420795 29333880 PMC5940523

[B48] Crowley N. A. Kash T. L. (2015). Kappa Opioid Receptor Signaling in the Brain: Circuitry and Implications for Treatment. Prog. Neuropsychopharmacol. Biol. Psychiatry 62, 51–60. 10.1016/j.pnpbp.2015.01.001 25592680 PMC4465498

[B49] Cunha-Oliveira T. Rego A. C. Oliveira C. R. (2008). Cellular and Molecular Mechanisms Involved in the Neurotoxicity of Opioid and Psychostimulant Drugs. Brain Res. Rev. 58 (1), 192–208. 10.1016/j.brainresrev.2008.03.002 18440072

[B50] Dai Z. Chu H. Ma J. Yan Y. Zhang X. Liang Y. (2018). The Regulatory Mechanisms and Therapeutic Potential of microRNAs: from Chronic Pain to Morphine Tolerance. Front. Mol. Neurosci. 11, 80. 10.3389/fnmol.2018.00080 29615865 PMC5864932

[B51] Dalley J. W. Lääne K. Pena Y. Theobald D. E. Everitt B. J. Robbins T. W. (2005). Attentional and Motivational Deficits in Rats Withdrawn from Intravenous Self-Administration of Cocaine or Heroin. Psychopharmacology (Berl) 182, 579–587. 10.1007/s00213-005-0107-3 16047195

[B52] Damghani F. Bigdeli I. Miladi-Gorji H. Fadaei A. (2016). Swimming Exercise Attenuates Psychological Dependence and Voluntary Methamphetamine Consumption in Methamphetamine Withdrawn Rats. Iran J. Basic Med. Sci. 19 (6), 594–600. 10.22038/ijbms.2016.7126 27482339 PMC4951597

[B53] Dao A. N. Beacher N. J. Mayr V. Montemarano A. Hammer S. West M. O. (2021). Chronic Fentanyl Self-Administration Generates a Shift toward Negative Affect in Rats during Drug Use. Brain Sci. 11 (8), 1064. 10.3390/brainsci11081064 34439683 PMC8394963

[B54] Darcq E. Kieffer B. L. (2018). Opioid Receptors: Drivers to Addiction? Nat. Rev. Neurosci. 19 (8), 499–514. 10.1038/s41583-018-0028-x 29934561

[B55] Das P. Horton R. (2019). The Global Drug Problem: Change but Not Progression. Lancet 394 (10208), 1488–1490. 10.1016/s0140-6736(19)32275-5 31657726

[B56] De Vito M. J. Wagner G. C. (1989). Methamphetamine-induced Neuronal Damage: a Possible Role for Free Radicals. Neuropharmacology 28 (10), 1145–1150. 10.1016/0028-3908(89)90130-5 2554183

[B57] Dean O. M. Bush A. I. Copolov D. L. Kohlmann K. Jeavons S. Schapkaitz I. (2012). Effects of N-Acetyl Cysteine on Cognitive Function in Bipolar Disorder. Psychiatry Clin. Neurosci. 66 (6), 514–517. 10.1111/j.1440-1819.2012.02392.x 23066769

[B58] Dezman Z. Schwartz B. Billing A. Massey E. Artigiani E. E. Factor J. (2020). Notes from the Field: High Prevalence of Fentanyl Detected by the Maryland Emergency Department Drug Surveillance System - Baltimore, Maryland, 2019. MMWR Morb Mortal Wkly Rep. 69 (23), 724–726. 10.15585/mmwr.mm6923a3 32525849 PMC7315791

[B59] Di Chiara G. Imperato A. (1988). Drugs Abused by Humans Preferentially Increase Synaptic Dopamine Concentrations in the Mesolimbic System of Freely Moving Rats. Proc. Natl. Acad. Sci. U S A. 85, 5274–5278. 10.1073/pnas.85.14.5274 2899326 PMC281732

[B60] Downey K. K. Helmus T. C. Schuster C. R. (2000). Treatment of Heroin-dependent Poly-Drug Abusers with Contingency Management and Buprenorphine Maintenance. Exp. Clin. Psychopharmacol. 8 (2), 176–184. 10.1037//1064-1297.8.2.176 10843300

[B61] Dugosh K. Abraham A. Seymour B. McLoyd K. Chalk M. Festinger D. (2016). A Systematic Review on the Use of Psychosocial Interventions in Conjunction with Medications for the Treatment of Opioid Addiction. J. Addict. Med. 10 (2), 93–103. 10.1097/adm.0000000000000193 26808307 PMC4795974

[B62] Duttaroy A. Yoburn B. C. (2000). *In Vivo* regulation of Mu-Opioid Receptor Density and Gene Expression in CXBK and Outbred Swiss Webster Mice. Synapse 37 (2), 118–124. 10.1002/1098-2396(200008)37:2<118::AID-SYN6>3.0.CO;2-2 10881033

[B63] Ebrahimi M. Mousavi S. R. Toussi A. G. Reihani H. Bagherian F. (2015). Comparing the Therapeutic Effectiveness of N-Acetylcysteine with the Combination of N-Acetyl Cysteine and Cimetidine in Acute Acetaminophen Toxicity: a Double-Blinded Clinical Trial. Electron. Physician 7 (6), 1310–1317. 10.14661/1310 26516435 PMC4623788

[B64] Eddy N. B. Isbell H. (1959). Addiction Liability and Narcotics Control. Public Health Rep. 74, 755–763. 10.2307/4590569 13819253 PMC1929309

[B65] Ellis M. S. Kasper Z. A. Cicero T. J. (2018). Twin Epidemics: the Surging Rise of Methamphetamine Use in Chronic Opioid Users. Drug Alcohol Depend 193, 14–20. 10.1016/j.drugalcdep.2018.08.029 30326396

[B66] Erbs E. Faget L. Scherrer G. Matifas A. Filliol D. Vonesch J. L. (2015). A Mu-delta Opioid Receptor Brain Atlas Reveals Neuronal Co-occurrence in Subcortical Networks. Brain Struct. Funct. 220, 677–702. 10.1007/s00429-014-0717-9 24623156 PMC4341027

[B67] Etaee F. Rezvani-Kamran A. Taheri M. Omidi G. Hasanein P. Komaki A. (2019). Comparing the Antinociceptive Effects of Methamphetamine, Buprenorphine, or Both after Chronic Treatment and Withdrawal in Male Rats. Basic Clin. Neurosci. 10, 313–322. 10.32598/bcn.9.10.160 32231768 PMC7101515

[B68] European Monitoring Centre for Drugs and Drug Addiction (EMCDDA) (2018). European Drug Report 2018. 10.2810/800331

[B69] Ezeomah C. Cunningham K. A. Stutz S. J. Fox R. G. Bukreyeva N. Dineley K. T. (2020). Fentanyl Self-Administration Impacts Brain Immune Responses in Male Sprague-Dawley Rats. Brain Behav. Immun. 87, 725–738. 10.1016/j.bbi.2020.03.003 32165150

[B70] Farhadian M. Akbarfahimi M. Hassani Abharian P. Hosseini S. G. Shokri S. (2017). Assessment of Executive Functions in Methamphetamine-Addicted Individuals: Emphasis on Duration of Addiction and Abstinence. Basic Clin. Neurosci. 8, 147. 10.18869/nirp.bcn.8.2.147 28539999 PMC5440924

[B71] Fitzpatrick R. E. Rubenis A. J. Lubman D. I. Verdejo-Garcia A. (2020). Cognitive Deficits in Methamphetamine Addiction: Independent Contributions of Dependence and Intelligence. Drug Alcohol Depend 209, 107891. 10.1016/j.drugalcdep.2020.107891 32061948

[B72] Fletcher J. E. Sebel P. S. Murphy M. R. Mick S. A. Fein S. (1991). Comparison of Ocfentanil and Fentanyl as Supplements to General Anesthesia. Anesth. Analg 73 (5), 622–626. 10.1213/00000539-199111000-00019 1952145

[B73] Fujii K. Koshidaka Y. Adachi M. Takao K. (2019). Effects of Chronic Fentanyl Administration on Behavioral Characteristics of Mice. Neuropsychopharmacol. Rep. 39 (1), 17–35. 10.1002/npr2.12040 30506634 PMC7292323

[B74] García-Cabrerizo R. García-Fuster M. J. (2019). Methamphetamine Binge Administration Dose-Dependently Enhanced Negative Affect and Voluntary Drug Consumption in Rats Following Prolonged Withdrawal: Role of Hippocampal FADD. Addict. Biol. 24 (2), 239–250. 10.1111/adb.12593 29282816

[B75] García-Fuster M. J. Ferrer-Alcón M. Miralles A. García-Sevilla J. s. A. (2003). Modulation of Fas Receptor Proteins and Dynamin during Opiate Addiction and Induction of Opiate Withdrawal in Rat Brain. Naunyn-Schmiedeberg's Arch. Pharmacol. 368, 421–431. 10.1007/s00210-003-0801-9 14530904

[B76] George P. Vicknasingam B. Thurairajasingam S. Ramasamy P. Mohd Yusof H. Yasin M. A. B. M. (2018). Methadone Complications Amongst Opioid-dependent Patients in Malaysia: A Case Series. Drug Alcohol. Rev. 37 (1), 147–151. 10.1111/dar.12456 27859761

[B77] Georgiou P. Zanos P. Garcia-Carmona J. A. Hourani S. Kitchen I. Laorden M. L. (2016). Methamphetamine Abstinence Induces Changes in μ-opioid Receptor, Oxytocin and CRF Systems: Association with an Anxiogenic Phenotype. Neuropharmacology 105, 520–532. 10.1016/j.neuropharm.2016.02.012 26896754

[B78] Gerrits M. A. Lesscher H. B. van Ree J. M. (2003). Drug Dependence and the Endogenous Opioid System. Eur. Neuropsychopharmacol. 13 (6), 424–434. 10.1016/j.euroneuro.2003.08.003 14636958

[B79] Gladden R. M. O'Donnell J. Mattson C. L. Seth P. (2019). Changes in Opioid-Involved Overdose Deaths by Opioid Type and Presence of Benzodiazepines, Cocaine, and Methamphetamine - 25 States, July-December 2017 to January-June 2018. MMWR Morb. Mortal. Wkly. Rep. 68, 737–744. 10.15585/mmwr.mm6834a2 PMC6715260 31465320

[B80] Glick S. N. Klein K. S. Tinsley J. Golden M. R. (2021). Increasing Heroin-Methamphetamine (Goofball) Use and Related Morbidity Among Seattle Area People Who Inject Drugs. Am. J. Addict. 30 (2), 183–191. 10.1111/ajad.13115 33301230 PMC8629025

[B81] Goldberg S. R. Schuster C. R. (1967). Conditioned Suppression by a Stimulus Associated with Nalorphine in Morphine-dependent Monkeys. J. Exp. Anal. Behav. 10 (3), 235–242. 10.1901/jeab.1967.10-235 4964349 PMC1338276

[B82] Grace P. M. Maier S. F. Watkins L. R. (2015). Opioid-induced central Immune Signaling: Implications for Opioid Analgesia. Headache 55 (4), 475–489. 10.1111/head.12552 25833219 PMC4402135

[B83] Grant J. E. Odlaug B. L. Kim S. W. (2010). A Double-Blind, Placebo-Controlled Study of N-Acetyl Cysteine Plus Naltrexone for Methamphetamine Dependence. Eur. Neuropsychopharmacol. 20 (11), 823–828. 10.1016/j.euroneuro.2010.06.018 20655182

[B84] Groman S. M. Lee B. Seu E. James A. S. Feiler K. Mandelkern M. A. (2012). Dysregulation of D₂-mediated Dopamine Transmission in Monkeys after Chronic Escalating Methamphetamine Exposure. J. Neurosci. 32 (17), 5843–5852. 10.1523/JNEUROSCI.0029-12.2012 22539846 PMC3353813

[B85] Gu J. Lau J. T. Xu H. Zhong Y. Hao Y. Zhao Y. (2013). A Randomized Controlled Trial to Evaluate the Relative Efficacy of the Addition of a Psycho-Social Intervention to Standard-Of-Care Services in Reducing Attrition and Improving Attendance Among First-Time Users of Methadone Maintenance Treatment in China. AIDS Behav. 17 (6), 2002–2010. 10.1007/s10461-012-0393-9 23413126

[B86] Hämmig R. Kemter A. Strasser J. von Bardeleben U. Gugger B. Walter M. (2016). Use of Microdoses for Induction of Buprenorphine Treatment with Overlapping Full Opioid Agonist Use: the Bernese Method. Subst. Abuse Rehabil. 7, 99–105. 10.2147/sar.s109919 27499655 PMC4959756

[B87] Harris G. C. Aston-Jones G. (2003). Critical Role for Ventral Tegmental Glutamate in Preference for a Cocaine-Conditioned Environment. Neuropsychopharmacology 28 (1), 73–76. 10.1038/sj.npp.1300011 12496942

[B88] Härtel-Petri R. Krampe-Scheidler A. Braunwarth W. D. Havemann-Reinecke U. Jeschke P. Looser W. (2017). Evidence-based Guidelines for the Pharmacologic Management of Methamphetamine Dependence, Relapse Prevention, Chronic Methamphetamine-Related, and Comorbid Psychiatric Disorders in post-acute Settings. Pharmacopsychiatry 50 (03), 96–104. 10.1055/s-0043-105500 28445899

[B89] Hayhurst C. J. Durieux M. E. (2016). Differential Opioid Tolerance and Opioid-Induced Hyperalgesia: a Clinical Reality. Anesthesiology 124 (2), 483–488. 10.1097/aln.0000000000000963 26594912

[B90] Hedegaard H. Minino A. Warner M. (2020). Drug Overdose Deaths in the United States. 1999-2018 (NCHS Data Brief No. 356). U.S. Department of Health and Human Services. 32487285

[B91] Heinzerling K. G. Swanson A. N. Kim S. Cederblom L. Moe A. Ling W. (2010). Randomized, Double-Blind, Placebo-Controlled Trial of Modafinil for the Treatment of Methamphetamine Dependence. Drug Alcohol Depend 109 (1-3), 20–29. 10.1016/j.drugalcdep.2009.11.023 20092966 PMC2875545

[B92] Hikin L. Smith P. R. Ringland E. Hudson S. Morley S. R. (2018). Multiple Fatalities in the North of England Associated with Synthetic Fentanyl Analogue Exposure: Detection and Quantitation a Case Series from Early 2017. Forensic Sci. Int. 282, 179–183. 10.1016/j.forsciint.2017.11.036 29216524

[B93] Hosseini M.-J. Askari Sadat-Mahaleh S. Ghavimi H. (2021). Selegiline Alleviates the Depressive-like Behaviors of Methamphetamine Withdrawal Syndrome through Modulating Mitochondrial Function and Energy Hemostasis. Pharm. Sci.. 10.34172/PS.2021.53

[B94] Hser Y. I. Li J. Jiang H. Zhang R. Du J. Zhang C. (2011). Effects of a Randomized Contingency Management Intervention on Opiate Abstinence and Retention in Methadone Maintenance Treatment in China. Addiction 106 (10), 1801–1809. 10.1111/j.1360-0443.2011.03490.x 21793958 PMC3174353

[B95] Ihongbe T. O. Masho S. W. (2016). Prevalence, Correlates and Patterns of Heroin Use Among Young Adults in the United States. Addict. Behav. 63, 74–81. 10.1016/j.addbeh.2016.07.003 27424167

[B96] Iman I. N. Ahmad N. A. Z. Mohd Yusof N. A. Talib U. N. Norazit A. Kumar J. (2021). Mitragynine (Kratom)-Induced Cognitive Impairments in Mice Resemble Δ9-THC and Morphine Effects: Reversal by Cannabinoid CB1 Receptor Antagonism. Front. Pharmacol. 12, 708055. 10.3389/fphar.2021.708055 34603022 PMC8481666

[B97] International Narcotics Control Board (INCB) (2013). Report of the International Narcotics Control Board for 2013. (E/INCB/2013/1). Available at: https://www.incb.org/documents/Publications/AnnualReports/AR2013/English/AR_2013_E.pdf .

[B98] Iudicello J. E. Woods S. P. Vigil O. Cobb Scott J. Cherner M. Heaton R. K. (2010). Longer Term Improvement in Neurocognitive Functioning and Affective Distress Among Methamphetamine Users Who Achieve Stable Abstinence. J. Clin. Exp. Neuropsychol. 32 (7), 704–718. 10.1080/13803390903512637 20198527 PMC2911490

[B99] Jacobs D. F. (1986). A General Theory of Addictions: A New Theoretical Model. J. Gambling Stud. 2, 15–31. 10.1007/BF01019931

[B100] Jacobskind J. S. Rosinger Z. J. Brooks M. L. Zuloaga D. G. (2019). Stress-induced Neural Activation Is Altered during Early Withdrawal from Chronic Methamphetamine. Behav. Brain Res. 366, 67–76. 10.1016/j.bbr.2019.03.034 30902659

[B101] Jalali F. Hashemi S. F. Hasani A. (2018). The Effectiveness of Cognitive-Behavioral Group Therapy in Reducing Craving Among Methamphetamine Abusers Living with HIV/AIDS. J. Drug Abuse 04 (2), 0. 10.21767/2471-853X.100076

[B102] Jia S. W. Wang W. Liu Y. Wu Z. M. (2005). Neuroimaging Studies of Brain Corpus Striatum Changes Among Heroin-dependent Patients Treated with Herbal Medicine, U'finer Capsule. Addict. Biol. 10 (3), 293–297. 10.1080/13556210500222456 16109593

[B103] Jia W. Liu R. Shi J. Wu B. Dang W. Du Y. (2013). Differential Regulation of MAPK Phosphorylation in the Dorsal hippocampus in Response to Prolonged Morphine Withdrawal-Induced Depressive-like Symptoms in Mice. PloS one 8 (6), e66111. 10.1371/journal.pone.0066111 23823128 PMC3688859

[B104] Jones C. M. Compton W. M. Mustaquim D. (2020). Patterns and Characteristics of Methamphetamine Use Among Adults - United States, 2015-2018. MMWR Morb Mortal Wkly Rep. 69 (12), 317–323. 10.15585/mmwr.mm6912a1 32214077 PMC7725509

[B105] Jones C. M. Logan J. Gladden R. M. Bohm M. K. (2015). Vital Signs: Demographic and Substance Use Trends Among Heroin Users - United States, 2002-2013. MMWR Morb Mortal Wkly Rep. 64 (26), 719–725. 10.1111/add.14812 26158353 PMC4584844

[B106] Jones H. W. Dean A. C. Price K. A. London E. D. (2016). Increased Self-Reported Impulsivity in Methamphetamine Users Maintaining Drug Abstinence. Am. J. Drug Alcohol. Abuse 42 (5), 500–506. 10.1080/00952990.2016.1192639 27398730 PMC5055455

[B107] Kaka G. Rahmanzade R. Safee F. Haghparast A. (2014). Naloxone Induces Frequent Jumping after Chronic Morphine and Methamphetamine Co-administration in Rats. Basic Clin. Neurosci. 5, 42–47. 25436083 PMC4202602

[B108] Kalant H. (2010). What Neurobiology Cannot Tell Us about Addiction. Addiction 105 (5), 780–789. 10.1111/j.1360-0443.2009.02739.x 19919596

[B109] Kalechstein A. D. Newton T. F. Green M. (2003). Methamphetamine Dependence Is Associated with Neurocognitive Impairment in the Initial Phases of Abstinence. J. Neuropsychiatry Clin. Neurosci. 15 (2), 215–220. 10.1176/jnp.15.2.215 12724464

[B110] Kampman K. Jarvis M. (2015). American Society of Addiction Medicine (ASAM) National Practice Guideline for the Use of Medications in the Treatment of Addiction Involving Opioid Use. J. Addict. Med. 9 (5), 358–367. 10.1097/ADM.0000000000000166 26406300 PMC4605275

[B111] Kariisa M. Scholl L. Wilson N. Seth P. Hoots B. (2019). Drug Overdose Deaths Involving Cocaine and Psychostimulants with Abuse Potential - United States, 2003-2017. MMWR Morb. Mortal. Wkly. Rep. 68, 388–395. 10.15585/mmwr.mm6817a3 31048676 PMC6541315

[B112] Karila L. Weinstein A. Aubin H. J. Benyamina A. Reynaud M. Batki S. L. (2010). Pharmacological Approaches to Methamphetamine Dependence: a Focused Review. Br. J. Clin. Pharmacol. 69 (6), 578–592. 10.1111/j.1365-2125.2010.03639.x 20565449 PMC2883750

[B113] Kecojevic A. Corliss H. L. Lankenau S. E. (2015). Motivations for Prescription Drug Misuse Among Young Men Who Have Sex with Men (YMSM) in Philadelphia. Int. J. Drug Pol. 26 (8), 764–771. 10.1016/j.drugpo.2015.03.010 PMC4499499 25936445

[B114] Kelty E. Joyce D. Hulse G. (2019). A Retrospective Cohort Study of Mortality Rates in Patients with an Opioid Use Disorder Treated with Implant Naltrexone, Oral Methadone or Sublingual Buprenorphine. Am. J. Drug Alcohol. Abuse 45 (3), 285–291. 10.1080/00952990.2018.1545131 30848965

[B115] Kieres A. K. Hausknecht K. A. Farrar A. M. Acheson A. De Wit H. Richards J. B. (2004). Effects of Morphine and Naltrexone on Impulsive Decision Making in Rats. Psychopharmacology (Berl) 173 (1), 167–174. 10.1007/s00213-003-1697-2 14752586

[B116] Kiluk B. D. Yip S. W. De Vito E. E. Carroll K. M. Sofuoglu M. (2019). Anhedonia as a Key Clinical Feature in the Maintenance and Treatment of Opioid Use Disorder. Clin. Psychol. Sci. 7 (6), 1190–1206. 10.1177/2167702619855659 32042509 PMC7009780

[B117] Kimbrough A. Kallupi M. Smith L. C. Simpson S. Collazo A. George O. (2021). Characterization of the Brain Functional Architecture of Psychostimulant Withdrawal Using Single-Cell Whole-Brain Imaging. Eneuro 8. 10.1523/ENEURO.0208-19.2021 PMC8570684 34580158

[B118] Kirby K. N. Petry N. M. (2004). Heroin and Cocaine Abusers Have Higher Discount Rates for Delayed Rewards Than Alcoholics or Non-drug-using Controls. Addiction 99 (4), 461–471. 10.1111/j.1360-0443.2003.00669.x 15049746

[B119] Kish S. J. Kalasinsky K. S. Derkach P. Schmunk G. A. Guttman M. Ang L. (2001). Striatal Dopaminergic and Serotonergic Markers in Human Heroin Users. Neuropsychopharmacology 24 (5), 561–567. 10.1016/S0893-133X(00)00209-8 11282256

[B120] Klenowski P. Morgan M. Bartlett S. E. (2015). The Role of δ-opioid Receptors in Learning and Memory Underlying the Development of Addiction. Br. J. Pharmacol. 172, 297–310. 10.1111/bph.12618 24641428 PMC4292947

[B121] Koob G. F. Buck C. L. Cohen A. Edwards S. Park P. E. Schlosburg J. E. (2014). Addiction as a Stress Surfeit Disorder. Neuropharmacology 76, 370–382. 10.1016/j.neuropharm.2013.05.024 23747571 PMC3830720

[B122] Koob G. F. Le Moal M. (2008). Addiction and the Brain Antireward System. Annu. Rev. Psychol. 59, 29–53. 10.1146/annurev.psych.59.103006.093548 18154498

[B123] Kouimtsidis C. Reynolds M. Coulton S. Drummond C. (2012). How Does Cognitive Behaviour Therapy Work with Opioid-dependent Clients? Results of the UKCBTMM Study. Drugs Educ. Prev. Pol. 19 (3), 253–258. 10.3109/09687637.2011.579194

[B124] Kras M. Youssef G. J. Garfield J. B. B. Yücel M. Lubman D. I. Stout J. C. (2018). Relationship between Measures of Impulsivity in Opioid-dependent Individuals. Personal. Individual Differences 120, 133–137. 10.1016/j.paid.2017.08.001

[B125] Krasnova I. N. Justinova Z. Ladenheim B. Jayanthi S. McCoy M. T. Barnes C. (2010). Methamphetamine Self-Administration Is Associated with Persistent Biochemical Alterations in Striatal and Cortical Dopaminergic Terminals in the Rat. PloS one 5 (1), e8790. 10.1371/journal.pone.0008790 20098750 PMC2808335

[B126] Kravitz A. V. Tye L. D. Kreitzer A. C. (2012). Distinct Roles for Direct and Indirect Pathway Striatal Neurons in Reinforcement. Nat. Neurosci. 15, 816–818. 10.1038/nn.3100 22544310 PMC3410042

[B127] Krupitsky E. Blokhina E. Zvartau E. Verbitskaya E. Lioznov D. Yaroslavtseva T. (2019). Slow-release Naltrexone Implant versus Oral Naltrexone for Improving Treatment Outcomes in People with HIV Who Are Addicted to Opioids: a Double-Blind, Placebo-Controlled, Randomised Trial. Lancet HIV 6 (4), e221–e229. 10.1016/s2352-3018(18)30362-x 30880163 PMC6529232

[B128] Kuczyńska K. Grzonkowski P. Kacprzak Ł. Zawilska J. B. (2018). Abuse of Fentanyl: An Emerging Problem to Face. Forensic Sci. Int. 289, 207–214. 10.1016/j.forsciint.2018.05.042 29902699

[B129] Kumar J. Hapidin H. Bee Y. T. Ismail Z. (2013). Effects of the mGluR5 Antagonist MPEP on Ethanol Withdrawal Induced Anxiety-like Syndrome in Rats. Behav. Brain Funct. 9 (1), 43–13. 10.1186/1744-9081-9-43 24279870 PMC4222772

[B130] Kumar J. Hapidin H. Get Bee Y. T. Ismail Z. (2016). The Effects of Acute Ethanol Administration on Ethanol Withdrawal-Induced Anxiety-like Syndrome in Rats: a Biochemical Study. Alcohol 50, 9–17. 10.1016/j.alcohol.2015.10.001 26626323

[B131] Kumar P. (2010). Use of Oral Methadone as an Analgesic: Review of the Cardiotoxic Side Effects. Clin. Med. Insights: Ther. 2, S3579. 10.4137/cmt.s3579

[B132] Lamb R. J. Preston K. L. Schindler C. W. Meisch R. A. Davis F. Katz J. L. (1991). The Reinforcing and Subjective Effects of Morphine in post-addicts: a Dose-Response Study. J. Pharmacol. Exp. Ther. 259, 1165–1173. 1762068

[B133] Lan K.-C. Liu S.-H. Lin-Shiau S.-Y. Chang A. (2015). Clinical Manifestations of Combined Methamphetamine with Morphine and Their Effects on Brain Dopamine and 5-hydroxytryptamine Release in Mice. J. Med. Sci. 35, 194. 10.4103/1011-4564.167740

[B134] Lan K. C. Chang A. C. Liu S. H. Ho I. K. Lin-Shiau S. Y. (2009). Enhancing Effects of Morphine on Methamphetamine-Induced Reinforcing Behavior and its Association with Dopamine Release and Metabolism in Mice. J. Neurochem. 109 (2), 382–392. 10.1111/j.1471-4159.2009.05998.x 19245664

[B135] Lankenau S. E. Teti M. Silva K. Jackson Bloom J. Harocopos A. Treese M. (2012). Initiation into Prescription Opioid Misuse Amongst Young Injection Drug Users. Int. J. Drug Pol. 23 (1), 37–44. 10.1016/j.drugpo.2011.05.014 PMC3196821 21689917

[B136] Laurent V. Morse A. K. Balleine B. W. (2015). The Role of Opioid Processes in Reward and Decision-Making. Br. J. Pharmacol. 172, 449–459. 10.1111/bph.12818 24930675 PMC4292959

[B137] Le Merrer J. Becker J. A. Befort K. Kieffer B. L. (2009). Reward Processing by the Opioid System in the Brain. Physiol. Rev. 89, 1379–1412. 10.1152/physrev.00005.2009 19789384 PMC4482114

[B138] Ledberg A. (2017). Mortality Related to Methadone Maintenance Treatment in Stockholm, Sweden, during 2006-2013. J. Subst. Abuse Treat. 74, 35–41. 10.1016/j.jsat.2016.12.005 28132698

[B139] Lentine K. L. Yuan H. Tuttle-Newhall J. E. Xiao H. Chawa V. Axelrod D. (2015). Quantifying Prognostic Impact of Prescription Opioid Use before Kidney Transplantation through Linked Registry and Pharmaceutical Claims Data. Transplantation 99 (1), 187–196. 10.1097/tp.0000000000000248 25531895

[B140] Li Z. Luan W. Chen Y. Chen M. Dong Y. Lai B. (2011). Chronic Morphine Treatment Switches the Effect of Dopamine on Excitatory Synaptic Transmission from Inhibition to Excitation in Pyramidal Cells of the Basolateral Amygdala. J. Neurosci. 31 (48), 17527–17536. 10.1523/jneurosci.3806-11.2011 22131414 PMC6623831

[B141] Li Z. Qi Y. Liu K. Cao Y. Zhang H. Song C. (2021). Effect of Chaihu-Jia-Longgu-Muli Decoction on Withdrawal Symptoms in Rats with Methamphetamine-Induced Conditioned Place Preference. Biosci. Rep. 41. 10.1042/bsr20211376 PMC8380915 34355745

[B142] Liang X. Liu R. Chen C. Ji F. Li T. (2016). Opioid System Modulates the Immune Function: a Review. Transl Perioper. Pain Med. 1 (1), 5–13. 10.1002/btm2.10014 PMC4790459 26985446

[B143] Liu C. C. Fang C. P. Liu T. H. Kuo H. W. Liu S. C. Wang S. C. (2018). APBB2 Is Associated with Amphetamine Use and Plasma Beta-Amyloids in Patients Receiving Methadone Maintenance Treatment. Prog. Neuropsychopharmacol. Biol. Psychiatry 83, 92–98. 10.1016/j.pnpbp.2018.01.008 29330135

[B144] Liu J. Liang J. Qin W. Tian J. Yuan K. Bai L. (2009). Dysfunctional Connectivity Patterns in Chronic Heroin Users: an fMRI Study. Neurosci. Lett. 460 (1), 72–77. 10.1016/j.neulet.2009.05.038 19450664

[B145] Liu N. Rockhold R. W. Ho I. K. (1999). Electrical Stimulation of Nucleus Paragigantocellularis Induces Opioid Withdrawal-like Behaviors in the Rat. Pharmacol. Biochem. Behav. 62, 263–271. 10.1016/s0091-3057(98)00164-6 9972693

[B146] Ljungberg T. Apicella P. Schultz W. (1992). Responses of Monkey Dopamine Neurons during Learning of Behavioral Reactions. J. Neurophysiol. 67, 145–163. 10.1152/jn.1992.67.1.145 1552316

[B147] London E. D. Kohno M. Morales A. M. Ballard M. E. (2015). Chronic Methamphetamine Abuse and Corticostriatal Deficits Revealed by Neuroimaging. Brain Res. 1628, 174–185. 10.1016/j.brainres.2014.10.044 25451127 PMC4418947

[B148] Luan X. Chen H. Qiu H. Shen H. Zhao K. Ren W. (2018). Association between Serum Malondialdehyde Levels and Depression during Early Methamphetamine Withdrawal. Neurosci. Lett. 687, 22–25. 10.1016/j.neulet.2018.09.021 30219487

[B149] Lutz P. E. Ayranci G. Chu-Sin-Chung P. Matifas A. Koebel P. Filliol D. (2014). Distinct Mu, delta, and Kappa Opioid Receptor Mechanisms Underlie Low Sociability and Depressive-like Behaviors during Heroin Abstinence. Neuropsychopharmacology 39, 2694–2705. 10.1038/npp.2014.126 24874714 PMC4207349

[B150] Lyons R. M. Yule A. M. Schiff D. Bagley S. M. Wilens T. E. (2019). Risk Factors for Drug Overdose in Young People: a Systematic Review of the Literature. J. Child. Adolesc. Psychopharmacol. 29 (7), 487–497. 10.1089/cap.2019.0013 31246496 PMC6727478

[B151] Lyoo I. K. Yoon S. Kim T. S. Lim S. M. Choi Y. Kim J. E. (2015). Predisposition to and Effects of Methamphetamine Use on the Adolescent Brain. Mol. Psychiatry 20 (12), 1516–1524. 10.1038/mp.2014.191 25666756 PMC5653271

[B152] Ma J. Sun X. J. Wang R. J. Wang T. Y. Su M. F. Liu M. X. (2018). Profile of Psychiatric Symptoms in Methamphetamine Users in China: Greater Risk of Psychiatric Symptoms with a Longer Duration of Use. Psychiatry Res. 262, 184–192. 10.1016/j.psychres.2018.02.017 29453037

[B153] Ma X. Qiu Y. Tian J. Wang J. Li S. Zhan W. (2015). Aberrant Default-Mode Functional and Structural Connectivity in Heroin-dependent Individuals. PLoS One 10 (4), e0120861. 10.1371/journal.pone.0120861 25859661 PMC4393019

[B154] Mancino M. J. Gentry B. W. Feldman Z. Mendelson J. Oliveto A. (2011). Characterizing Methamphetamine Withdrawal in Recently Abstinent Methamphetamine Users: a Pilot Field Study. Am. J. Drug Alcohol. Abuse 37 (2), 131–136. 10.3109/00952990.2010.543998 21219261 PMC3063384

[B155] Manzanedo C. Aguilar M. A. Rodríguez-Arias M. Miñarro J. (2005). Sensitization to the Rewarding Effects of Morphine Depends on Dopamine. Neuroreport 16, 201–205. 10.1097/00001756-200502080-00028 15671878

[B156] Marchei E. Pacifici R. Mannocchi G. Marinelli E. Busardò F. P. Pichini S. (2018). New Synthetic Opioids in Biological and Non-biological Matrices: A Review of Current Analytical Methods. Trac Trends Anal. Chem. 102, 1–15. 10.1016/j.trac.2018.01.007

[B157] Marsch L. A. Guarino H. Acosta M. Aponte-Melendez Y. Cleland C. Grabinski M. (2014). Web-based Behavioral Treatment for Substance Use Disorders as a Partial Replacement of Standard Methadone Maintenance Treatment. J. Subst. Abuse Treat. 46 (1), 43–51. 10.1016/j.jsat.2013.08.012 24060350 PMC3839618

[B158] Martinez D. Slifstein M. Broft A. Mawlawi O. Hwang D. R. Huang Y. (2003). Imaging Human Mesolimbic Dopamine Transmission with Positron Emission Tomography. Part II: Amphetamine-Induced Dopamine Release in the Functional Subdivisions of the Striatum. J. Cereb. Blood Flow Metab. 23 (3), 285–300. 10.1097/01.WCB.0000048520.34839.1A 12621304

[B159] Mateu-Gelabert P. Guarino H. Jessell L. Teper A. (2015). Injection and Sexual HIV/HCV Risk Behaviors Associated with Nonmedical Use of Prescription Opioids Among Young Adults in New York City. J. Subst. Abuse Treat. 48 (1), 13–20. 10.1016/j.jsat.2014.07.002 25124258 PMC4250325

[B160] McCann D. J. (2008). Potential of Buprenorphine/naltrexone in Treating Polydrug Addiction and Co-occurring Psychiatric Disorders. Clin. Pharmacol. Ther. 83 (4), 627–630. 10.1038/sj.clpt.6100503 18212797

[B161] McCann U. D. Wong D. F. Yokoi F. Villemagne V. Dannals R. F. Ricaurte G. A. (1998). Reduced Striatal Dopamine Transporter Density in Abstinent Methamphetamine and Methcathinone Users: Evidence from Positron Emission Tomography Studies with [11C]WIN-35,428. J. Neurosci. 18 (20), 8417–8422. 10.1523/jneurosci.18-20-08417.1998 9763484 PMC6792853

[B162] McConnell S. A. Brandner A. J. Blank B. A. Kearns D. N. Koob G. F. Vendruscolo L. F. (2021). Demand for Fentanyl Becomes Inelastic Following Extended Access to Fentanyl Vapor Self-Administration. Neuropharmacology 182, 108355. 10.1016/j.neuropharm.2020.108355 33091459 PMC7747488

[B163] McNeil R. Puri N. Boyd J. Mayer S. Hayashi K. Small W. (2020). Understanding Concurrent Stimulant Use Among People on Methadone: A Qualitative Study. Drug Alcohol. Rev. 39 (3), 209–215. 10.1111/dar.13049 32202009 PMC7302260

[B164] Melega W. P. Cho A. K. Harvey D. Laćan G. (2007). Methamphetamine Blood Concentrations in Human Abusers: Application to Pharmacokinetic Modeling. Synapse 61, 216–220. 10.1002/syn.20365 17230548

[B165] Melega W. P. Jorgensen M. J. Laćan G. Way B. M. Pham J. Morton G. (2008). Long-term Methamphetamine Administration in the Vervet Monkey Models Aspects of a Human Exposure: Brain Neurotoxicity and Behavioral Profiles. Neuropsychopharmacology 33 (6), 1441–1452. 10.1038/sj.npp.1301502 17625500

[B166] Menegas W. Akiti K. Amo R. Uchida N. Watabe-Uchida M. (2018). Dopamine Neurons Projecting to the Posterior Striatum Reinforce Avoidance of Threatening Stimuli. Nat. Neurosci. 21, 1421–1430. 10.1038/s41593-018-0222-1 30177795 PMC6160326

[B167] Meye F. J. van Zessen R. Smidt M. P. Adan R. A. Ramakers G. M. (2012). Morphine Withdrawal Enhances Constitutive μ-opioid Receptor Activity in the Ventral Tegmental Area. J. Neurosci. 32 (46), 16120–16128. 10.1523/JNEUROSCI.1572-12.2012 23152596 PMC6794017

[B168] Miladi-Gorji H. Rashidy-Pour A. Fathollahi Y. (2012). Anxiety Profile in Morphine-dependent and Withdrawn Rats: Effect of Voluntary Exercise. Physiol. Behav. 105 (2), 195–202. 10.1016/j.physbeh.2011.08.010 21871908

[B169] Miles S. W. Sheridan J. Russell B. Kydd R. Wheeler A. Walters C. (2013). Extended-release Methylphenidate for Treatment of Amphetamine/methamphetamine Dependence: a Randomized, Double-Blind, Placebo-Controlled Trial. Addiction 108 (7), 1279–1286. 10.1111/add.12109 23297867

[B170] Mileykovskiy B. Morales M. (2011). Duration of Inhibition of Ventral Tegmental Area Dopamine Neurons Encodes a Level of Conditioned Fear. J. Neurosci. 31, 7471–7476. 10.1523/jneurosci.5731-10.2011 21593330 PMC3153414

[B171] Minozzi S. Amato L. Vecchi S. Davoli M. Kirchmayer U. Verster A. (2011). Oral Naltrexone Maintenance Treatment for Opioid Dependence. Cochrane Database Syst. Rev. 2011 (2), CD001333. 10.1002/14651858.cd001333.pub4 16437431

[B172] Mishra A. Singh S. Shukla S. (2018). Physiological and Functional Basis of Dopamine Receptors and Their Role in Neurogenesis: Possible Implication for Parkinson's Disease. J. Exp. Neurosci. 12, 1179069518779829. 10.1177/1179069518779829 29899667 PMC5985548

[B173] Mizoguchi H. Yamada K. (2019). Methamphetamine Use Causes Cognitive Impairment and Altered Decision-Making. Neurochem. Int. 124, 106–113. 10.1016/j.neuint.2018.12.019 30611760

[B174] Moazen P. Azizi H. Salmanzadeh H. Semnanian S. (2018). Adolescent Morphine Exposure Induces Immediate and Long-Term Increases in Impulsive Behavior. Psychopharmacology (Berl) 235 (12), 3423–3434. 10.1007/s00213-018-5051-0 30350222

[B175] Moon M. Do K. S. Park J. Kim D. (2007). Memory Impairment in Methamphetamine Dependent Patients. Int. J. Neurosci. 117 (1), 1–9. 10.1080/00207450500535503 17365096

[B176] Mori T. Ito S. Namiki M. Suzuki T. Kobayashi S. Matsubayashi K. (2007). Involvement of Free Radicals Followed by the Activation of Phospholipase A2 in the Mechanism that Underlies the Combined Effects of Methamphetamine and Morphine on Subacute Toxicity or Lethality in Mice: Comparison of the Therapeutic Potential of Fullerene, Mepacrine, and Cooling. Toxicology 236 (3), 149–157. 10.1016/j.tox.2007.03.027 17553606

[B177] Mori T. Iwase Y. Saeki T. Iwata N. Murata A. Masukawa D. (2016). Differential Activation of Dopaminergic Systems in Rat Brain Basal Ganglia by Morphine and Methamphetamine. Neuroscience 322, 164–170. 10.1016/j.neuroscience.2016.01.043 26820597

[B178] Morley K. C. Cornish J. L. Faingold A. Wood K. Haber P. S. (2017a). Pharmacotherapeutic Agents in the Treatment of Methamphetamine Dependence. Expert Opin. Investig. Drugs 26 (5), 563–578. 10.1080/13543784.2017.1313229 28351169

[B179] Morley K. I. Ferris J. A. Winstock A. R. Lynskey M. T. (2017b). Polysubstance Use and Misuse or Abuse of Prescription Opioid Analgesics: a Multi-Level Analysis of International Data. Pain 158 (6), 1138–1144. 10.1097/j.pain.0000000000000892 28267061

[B180] Mousavi S. G. Sharbafchi M. R. Salehi M. Peykanpour M. Karimian Sichani N. Maracy M. (2015). The Efficacy of N-Acetylcysteine in the Treatment of Methamphetamine Dependence: a Double-Blind Controlled, Crossover Study. Arch. Iran Med. 18 (1), 28–33. 25556383

[B181] Namiki M. Mori T. Sawaguchi T. Ito S. Suzuki T. (2005). Underlying Mechanism of Combined Effect of Methamphetamine and Morphine on Lethality in Mice and Therapeutic Potential of Cooling. J. Pharmacol. Sci. 99 (2), 168–176. 10.1254/jphs.fpj05004x 16210775

[B182] National Anti-Drugs Agency (NADA) (2019). Annual Report National Anti-drugs Agency 2019. Available at: https://www.adk.gov.my/buku-laporan-tahunan-2019/ .

[B183] National Drug Intelligence Centre (NDIC) (2011). National Drug Threat Assessment. Available at: https://www.justice.gov/archive/ndic/pubs44/44849/44849p.pdf .

[B184] National Highway Traffic Safety Administration (2004). Drugs and Human Performance Fact Sheets. (DOT HS 809 725). Available at: https://www.nhtsa.gov/sites/nhtsa.gov/files/809725-drugshumanperformfs.pdf .

[B185] Negus S. S. Gatch M. B. Mello N. K. (1998). Effects of Mu Opioid Agonists Alone and in Combination with Cocaine and D-Amphetamine in Rhesus Monkeys Trained to Discriminate Cocaine. Neuropsychopharmacology 18, 325–338. 10.1016/s0893-133x(97)00163-2 9536446

[B186] Ogawa S. K. Watabe-Uchida M. (2017). Organization of Dopamine and Serotonin System: Anatomical and Functional Mapping of Monosynaptic Inputs Using Rabies Virus. Pharmacol. Biochem. Behav. 174, 9–22. 10.1016/j.pbb.2017.05.001 28476484

[B187] Olmstead M. C. Ouagazzal A. M. Kieffer B. L. (2009). Mu and delta Opioid Receptors Oppositely Regulate Motor Impulsivity in the Signaled Nose Poke Task. PLoS ONE 4, e4410. 10.1371/journal.pone.0004410 19198656 PMC2635474

[B188] Pakri Mohamed R. M. Kumar J. Ahmad S. U. Mohamed I. N. (2018). Novel Pharmacotherapeutic Approaches in Treatment of Alcohol Addiction. Curr. Drug Targets 19 (12), 1378–1390. 10.2174/1389450119666180523092534 29788886

[B189] Palamar J. J. Le A. Mateu-Gelabert P. (2018). Not just Heroin: Extensive Polysubstance Use Among US High School Seniors Who Currently Use Heroin. Drug Alcohol Depend 188, 377–384. 10.1016/j.drugalcdep.2018.05.001 29880271 PMC6198323

[B190] Palmer A. Scott N. Dietze P. Higgs P. (2020). Motivations for crystal Methamphetamine-Opioid Co-injection/co-use Amongst Community-Recruited People Who Inject Drugs: a Qualitative Study. Harm Reduct J. 17 (1), 14. 10.1186/s12954-020-00360-9 32106854 PMC7047412

[B191] Park S. W. Shen X. Tien L. T. Roman R. Ma T. (2011). Methamphetamine-induced Changes in the Striatal Dopamine Pathway in μ-opioid Receptor Knockout Mice. J. Biomed. Sci. 18 (1), 83–10. 10.1186/1423-0127-18-83 22074218 PMC3228795

[B192] Pattij T. Schetters D. Janssen M. C. Wiskerke J. Schoffelmeer A. N. (2009). Acute Effects of Morphine on Distinct Forms of Impulsive Behavior in Rats. Psychopharmacology (Berl) 205 (3), 489–502. 10.1007/s00213-009-1558-8 19436995 PMC2712067

[B193] Pellissier L. P. Pujol C. N. Becker J. A. J. Le Merrer J. (2016). Delta Opioid Receptors: Learning and Motivation. Handb Exp. Pharmacol. 247, 227–260. 10.1007/164_2016_89 28035528

[B194] Pergolizzi J. V. Jr Raffa R. B. Rosenblatt M. H. (2020). Opioid Withdrawal Symptoms, a Consequence of Chronic Opioid Use and Opioid Use Disorder: Current Understanding and Approaches to Management. J. Clin. Pharm. Ther. 45 (5), 892–903. 10.1111/jcpt.13114 31986228

[B195] Pirastu R. Fais R. Messina M. Bini V. Spiga S. Falconieri D. (2006). Impaired Decision-Making in Opiate-dependent Subjects: Effect of Pharmacological Therapies. Drug Alcohol Depend 83 (2), 163–168. 10.1016/j.drugalcdep.2005.11.008 16343811

[B196] Polcin D. L. Bond J. Korcha R. Nayak M. B. Galloway G. P. Evans K. (2014). Randomized Trial of Intensive Motivational Interviewing for Methamphetamine Dependence. J. Addict. Dis. 33 (3), 253–265. 10.1080/10550887.2014.950029 25115166 PMC4224618

[B197] Preston K. L. Jobes M. L. Phillips K. A. Epstein D. H. (2016). Real-time Assessment of Alcohol Drinking and Drug Use in Opioid-dependent Polydrug Users. Behav. Pharmacol. 27 (7), 579–584. 10.1097/FBP.0000000000000250 27579810 PMC5010032

[B198] Proebstl L. Krause D. Kamp F. Hager L. Manz K. Schacht-Jablonowsky M. (2019). Methamphetamine Withdrawal and the Restoration of Cognitive Functions - a Study over a Course of 6 Months Abstinence. Psychiatry Res. 281, 112599. 10.1016/j.psychres.2019.112599 31629302

[B199] Qiu Y. W. Han L. J. Lv X. F. Jiang G. H. Tian J. Z. Zhuo F. Z. (2011). Regional Homogeneity Changes in Heroin-dependent Individuals: Resting-State Functional MR Imaging Study. Radiology 261 (2), 551–559. 10.1148/radiol.11102466 21875854

[B200] Qiu Y. W. Jiang G. H. Su H. H. Lv X. F. Tian J. Z. Li L. M. (2013). The Impulsivity Behavior Is Correlated with Prefrontal Cortex gray Matter Volume Reduction in Heroin-dependent Individuals. Neurosci. Lett. 538, 43–48. 10.1016/j.neulet.2013.01.019 23352851

[B201] Rabin D. U. Tarbox H. K. Colburn L. Alfred K. C. (2017). Extended-Release and Long-Acting Opioid Analgesics Risk Evaluation and Mitigation Strategy (REMS):. J. Med. Regul. 103 (4), 7–16. 10.30770/2572-1852-103.4.7

[B202] Ramezany Yasuj S. Nourhashemi M. Keshavarzi S. Motaghinejad M. Motevalian M. (2019). Possible Role of Cyclic AMP Response Element Binding/brain-Derived Neurotrophic Factor Signaling Pathway in Mediating the Pharmacological Effects of Duloxetine against Methamphetamine Use-Induced Cognitive Impairment and Withdrawal-Induced Anxiety and Depression in Rats. Adv. Biomed. Res. 8, 11. 10.4103/abr.abr_34_18 30993081 PMC6425746

[B203] Ranaldi R. Wise R. A. (2000). Intravenous Self-Administration of Methamphetamine-Heroin (Speedball) Combinations under a Progressive-Ratio Schedule of Reinforcement in Rats. Neuroreport 11, 2621–2623. 10.1097/00001756-200008210-00003 10976931

[B204] Rapeli P. Kivisaari R. Autti T. Kähkönen S. Puuskari V. Jokela O. (2006). Cognitive Function during Early Abstinence from Opioid Dependence: a Comparison to Age, Gender, and Verbal Intelligence Matched Controls. Bmc Psychiatry 6 (1), 9–10. 10.1186/1471-244X-6-9 16504127 PMC1489929

[B205] Rawson R. Glasner S. Brecht M. L. Farabee D. (2021). A Randomized Comparison of 4 vs. 16 Weeks of Psychosocial Treatment for Stimulant Users. J. Subst. Abuse Treat. 124, 108274. 10.1016/j.jsat.2020.108274 33771278

[B206] Ren W. Luan X. Zhang J. Gutteea P. Cai Y. Zhao J. (2017). Brain-derived Neurotrophic Factor Levels and Depression during Methamphetamine Withdrawal. J. Affect Disord. 221, 165–171. 10.1016/j.jad.2017.06.017 28647666

[B207] Rezaei F. Emami M. Zahed S. Morabbi M. J. Farahzadi M. Akhondzadeh S. (2015). Sustained-release Methylphenidate in Methamphetamine Dependence Treatment: a Double-Blind and Placebo-Controlled Trial. Daru 23 (1), 2–8. 10.1186/s40199-015-0092-y 25588930 PMC4298048

[B208] Rezaeian L. Kalalian-Moghaddam H. Mohseni F. Khaksari M. Rafaiee R. (2020). Effects of Berberine Hydrochloride on Methamphetamine-Induced Anxiety Behaviors and Relapse in Rats. Iran J. Basic Med. Sci. 23 (11), 1480–1488. 10.22038/ijbms.2020.47285.10884 33235706 PMC7671426

[B209] Ridzwan I. E. Suhaimi M. S. Muhamad A. H. Kasmuri A. R. Mohamed Nazar N. I. Hashim R. (2018). “Low-dose Methamphetamine Addiction Induced Opioid Receptor Sensitization in Polydrug-dependent Mice,” in Regional Conference on Science, Technology and Social Sciences (RCSTSS 2016) (Singapore: Springer), 613–624. 10.1007/978-981-13-0074-5_59

[B210] Rigg K. K. Ibañez G. E. (2010). Motivations for Non-medical Prescription Drug Use: A Mixed Methods Analysis. J. Subst. Abuse Treat. 39 (3), 236–247. 10.1016/j.jsat.2010.06.004 20667680 PMC2937068

[B211] Robles E. Stitzer M. L. Strain E. C. Bigelow G. E. Silverman K. (2002). Voucher-based Reinforcement of Opiate Abstinence during Methadone Detoxification. Drug Alcohol Depend 65 (2), 179–189. 10.1016/S0376-8716(01)00160-0 11772479

[B212] Rothberg R. L. Stith K. (2018). Fentanyl: a Whole New World? J. L. Med Ethics 46 (2), 314–324. 10.1177/1073110518782937 30147007

[B213] Rowlett J. K. Platt D. M. Yao W. D. Spealman R. D. (2007). Modulation of Heroin and Cocaine Self-Administration by Dopamine D1- and D2-like Receptor Agonists in Rhesus Monkeys. J. Pharmacol. Exp. Ther. 321 (3), 1135–1143. 10.1124/jpet.107.120766 17351103

[B214] Ru Q. Xiong Q. Zhou M. Chen L. Tian X. Xiao H. (2019). Withdrawal from Chronic Treatment with Methamphetamine Induces Anxiety and Depression-like Behavior in Mice. Psychiatry Res. 271, 476–483. 10.1016/j.psychres.2018.11.072 30544074

[B215] Ruhm C. J. (2019). Nonopioid Overdose Death Rates Rose Almost as Fast as Those Involving Opioids, 1999-2016. Health Aff. 38, 1216–1224. 10.1377/hlthaff.2018.05522 31260365

[B216] Sabrini S. Wang G. Y. Lin J. C. Ian J. K. Curley L. E. (2019). Methamphetamine Use and Cognitive Function: A Systematic Review of Neuroimaging Research. Drug Alcohol Depend 194, 75–87. 10.1016/j.drugalcdep.2018.08.041 30414539

[B217] Sadat-Shirazi M. S. Zarrindast M. R. Daneshparvar H. Ziaie A. Fekri M. Abbasnezhad E. (2018). Alteration of Dopamine Receptors Subtypes in the Brain of Opioid Abusers: a Postmortem Study in Iran. Neurosci. Lett. 687, 169–176. 10.1016/j.neulet.2018.09.043 30268777

[B218] Salehi M. (2015). The Efficacy of N-Acetylcysteine in the Treatment of Methamphetamine Dependence: a Double-Blind Controlled, Crossover Study. Arch. Iranian Med. 18 (1), 28–33. 25556383

[B219] Saucier R. Wolfe D. Dasgupta N. (2018). Review of Case Narratives from Fatal Overdoses Associated with Injectable Naltrexone for Opioid Dependence. Drug Saf. 41 (10), 981–988. 10.1007/s40264-018-0653-3 29560596

[B220] Schottenfeld R. S. Chawarski M. C. Pakes J. R. Pantalon M. V. Carroll K. M. Kosten T. R. (2005). Methadone versus Buprenorphine with Contingency Management or Performance Feedback for Cocaine and Opioid Dependence. Am. J. Psychiatry 162 (2), 340–349. 10.1176/appi.ajp.162.2.340 15677600

[B221] Schwartz R. P. Kelly S. M. Mitchell S. G. Gryczynski J. O'Grady K. E. Jaffe J. H. (2017). When Does Methadone Treatment Reduce Arrest and Severity of Arrest Charges? an Analysis of Arrest Records. Drug Alcohol Depend 180, 385–390. 10.1016/j.drugalcdep.2017.08.025 28961545 PMC5667939

[B222] Seaman R. W. Collins G. T. Lordson C. (2021). Impact of Morphine Dependence and Withdrawal on the Reinforcing Effectiveness of Fentanyl, Cocaine, and Methamphetamine in Rats. Front. Pharmacol. 12, 1266. 10.3389/fphar.2021.691700 PMC8175987 34093214

[B223] Sekine Y. Iyo M. Ouchi Y. Matsunaga T. Tsukada H. Okada H. (2001). Methamphetamine-related Psychiatric Symptoms and Reduced Brain Dopamine Transporters Studied with PET. Am. J. Psychiatry 158 (8), 1206–1214. 10.1176/appi.ajp.158.8.1206 11481152

[B224] Sekine Y. Minabe Y. Ouchi Y. Takei N. Iyo M. Nakamura K. (2003). Association of Dopamine Transporter Loss in the Orbitofrontal and Dorsolateral Prefrontal Cortices with Methamphetamine-Related Psychiatric Symptoms. Am. J. Psychiatry 160 (9), 1699–1701. 10.1176/appi.ajp.160.9.1699 12944350

[B225] Shabani S. Schmidt B. Ghimire B. Houlton S. K. Hellmuth L. Mojica E. (2018). Depression-like Symptoms of Withdrawal in a Genetic Mouse Model of Binge Methamphetamine Intake. Genes, Brain Behav. 18, e12533. 10.1111/gbb.12533 30375183 PMC6399044

[B226] Shakiba K. Effatpanah M. Moradi A. (2018). Cognitive-behavioral Therapy for Methamphetamine Dependence Among Methadone-Maintained Patients. Iran J. Psychiatry Behav. Sci. 12 (2). 10.5812/ijpbs.63615

[B227] Shearer R. D. Howell B. A. Bart G. Winkelman T. N. A. (2020). Substance Use Patterns and Health Profiles Among US Adults Who Use Opioids, Methamphetamine, or Both, 2015-2018. Drug Alcohol Depend 214, 108162. 10.1016/j.drugalcdep.2020.108162 32652380 PMC8147519

[B228] Shi J. Zhao L. Y. Copersino M. L. Fang Y. X. Chen Y. Tian J. (2008). PET Imaging of Dopamine Transporter and Drug Craving during Methadone Maintenance Treatment and after Prolonged Abstinence in Heroin Users. Eur. J. Pharmacol. 579 (1-3), 160–166. 10.1016/j.ejphar.2007.09.042 17977528

[B229] Shi Z. Jagannathan K. Padley J. H. Wang A. L. Fairchild V. P. O'Brien C. P. (2021). The Role of Withdrawal in Mesocorticolimbic Drug Cue Reactivity in Opioid Use Disorder. Addict. Biol. 26 (4), e12977. 10.1111/adb.12977 33098179

[B230] Siefried K. J. Acheson L. S. Lintzeris N. Ezard N. (2020). Pharmacological Treatment of Methamphetamine/amphetamine Dependence: a Systematic Review. CNS drugs 34 (4), 337–365. 10.1007/s40263-020-00711-x 32185696 PMC7125061

[B231] Siegal H. A. Carlson R. G. Kenne D. R. Swora M. G. (2003). Probable Relationship between Opioid Abuse and Heroin Use. Am. Fam. Physician 67 (5), 942–945. 12643356

[B232] Silva K. Kecojevic A. Lankenau S. E. (2013). Perceived Drug Use Functions and Risk Reduction Practices Among High-Risk Nonmedical Users of Prescription Drugs. J. Drug Issues 43 (4), 483–496. 10.1177/0022042613491099 25477621 PMC4251706

[B233] Silveira M. L. Green V. R. Iannaccone R. Kimmel H. L. Conway K. P. (2019). Patterns and Correlates of Polysubstance Use Among US Youth Aged 15-17 Years: Wave 1 of the Population Assessment of Tobacco and Health (PATH) Study. Addiction 114 (5), 907–916. 10.1111/add.14547 30614093 PMC6609515

[B234] Silverstein S. M. Daniulaityte R. Martins S. S. Miller S. C. Carlson R. G. (2019). "Everything Is Not Right Anymore": Buprenorphine Experiences in an Era of Illicit Fentanyl. Int. J. Drug Pol. 74, 76–83. 10.1016/j.drugpo.2019.09.003 PMC6914257 31563098

[B235] Simon S. L. Dean A. C. Cordova X. Monterosso J. R. London E. D. (2010). Methamphetamine Dependence and Neuropsychological Functioning: Evaluating Change during Early Abstinence. J. Stud. Alcohol. Drugs 71 (3), 335–344. 10.15288/jsad.2010.71.335 20409426 PMC2859784

[B236] Singh D. Kamal W. M. Narayanan S. Vicknasingam B. (2020). Methamphetamine Use and Misconceptions Among Primary and Private Methadone Maintenance Treatment (MMT) Program Attendees in Klang Valley, Malaysia. J. Substance Use 25 (5), 528–534. 10.1080/14659891.2020.1738573

[B237] Sivaraman J. J. Greene S. B. Naumann R. B. Proescholdbell S. Ranapurwala S. I. Marshall S. W. (2021). Characterizing Opioid Overdoses Using Emergency Medical Services Data : A Case Definition Algorithm Enhanced by Machine Learning. Public Health Rep. 136, 62S–71S. 10.1177/00333549211026802 34726978 PMC8573782

[B238] Smith J. E. Co C. Coller M. D. Hemby S. E. Martin T. J. (2006). Self-administered Heroin and Cocaine Combinations in the Rat: Additive Reinforcing Effects-Supra-Additive Effects on Nucleus Accumbens Extracellular Dopamine. Neuropsychopharmacology 31 (1), 139–150. 10.1038/sj.npp.1300786 15956989 PMC4048550

[B239] Smout M. F. Longo M. Harrison S. Minniti R. Wickes W. White J. M. (2010). Psychosocial Treatment for Methamphetamine Use Disorders: A Preliminary Randomized Controlled Trial of Cognitive Behavior Therapy and Acceptance and Commitment Therapy. Subst. Abus 31 (2), 98–107. 10.1080/08897071003641578 20408061

[B240] Smyth B. P. Elmusharaf K. Cullen W. (2018). Opioid Substitution Treatment and Heroin Dependent Adolescents: Reductions in Heroin Use and Treatment Retention over Twelve Months. BMC Pediatr. 18 (1), 151–212. 10.1186/s12887-018-1137-4 29728088 PMC5936020

[B241] Solomon R. L. Corbit J. D. (1973). An Opponent-Process Theory of Motivation. II. Cigarette Addiction. J. Abnorm. Psychol. 81, 158–171. 10.1037/h0034534 4697797

[B242] Sontate K. V. Rahim Kamaluddin M. Naina Mohamed I. Mohamed R. M. P. Shaikh M. F. Kamal H. (2021). Alcohol, Aggression, and Violence: from Public Health to Neuroscience. Front. Psychol. 12, 699726. 10.3389/fpsyg.2021.699726 35002823 PMC8729263

[B243] Spagnolo P. A. Kimes A. Schwandt M. L. Shokri-Kojori E. Thada S. Phillips K. A. (2019). Striatal Dopamine Release in Response to Morphine: A [11C]Raclopride Positron Emission Tomography Study in Healthy Men. Biol. Psychiatry 86 (5), 356–364. 10.1016/j.biopsych.2019.03.965 31097294 PMC6699765

[B244] Spanagel R. Herz A. Shippenberg T. S. (1992). Opposing Tonically Active Endogenous Opioid Systems Modulate the Mesolimbic Dopaminergic Pathway. Proc. Natl. Acad. Sci. U S A. 89, 2046–2050. 10.1073/pnas.89.6.2046 1347943 PMC48593

[B245] Sprouse-Blum A. S. Smith G. Sugai D. Parsa F. D. (2010). Understanding Endorphins and Their Importance in Pain Management. Hawaii Med. J. 69 (3), 70–71. 20397507 PMC3104618

[B246] Stanley T. H. (2014). The Fentanyl story. J. Pain 15 (12), 1215–1226. 10.1016/j.jpain.2014.08.010 25441689

[B247] Steuer A. E. Williner E. Staeheli S. N. Kraemer T. (2017). Studies on the Metabolism of the Fentanyl-Derived Designer Drug Butyrfentanyl in Human *In Vitro* Liver Preparations and Authentic Human Samples Using Liquid Chromatography-High Resolution Mass Spectrometry (LC-HRMS). Drug Test. Anal. 9 (7), 1085–1092. 10.1002/dta.2111 27736030

[B248] Stevens J. P. Wall M. J. Novack L. Marshall J. Hsu D. J. Howell M. D. (2017). The Critical Care Crisis of Opioid Overdoses in the United States. Ann. Am. Thorac. Soc. 14, 1803–1809. 10.1513/annalsats.201701-022oc 28800256 PMC5802515

[B249] Stewart J. de Wit H. Eikelboom R. (1984). Role of Unconditioned and Conditioned Drug Effects in the Self-Administration of Opiates and Stimulants. Psychol. Rev. 91, 251–268. 10.1037/0033-295x.91.2.251 6571424

[B250] Stone A. C. Carroll J. J. Rich J. D. Green T. C. (2018). Methadone Maintenance Treatment Among Patients Exposed to Illicit Fentanyl in Rhode Island: Safety, Dose, Retention, and Relapse at 6 Months. Drug Alcohol Depend 192, 94–97. 10.1016/j.drugalcdep.2018.07.019 30243145 PMC10246725

[B251] Stotts A. L. Green C. Masuda A. Grabowski J. Wilson K. Northrup T. F. (2012). A Stage I Pilot Study of Acceptance and Commitment Therapy for Methadone Detoxification. Drug Alcohol Depend 125 (3), 215–222. 10.1016/j.drugalcdep.2012.02.015 22425411 PMC3386351

[B252] Su H. Zhang J. Ren W. Xie Y. Tao J. Zhang X. (2017). Anxiety Level and Correlates in Methamphetamine-dependent Patients during Acute Withdrawal. Medicine (Baltimore) 96 (15), e6434. 10.1097/md.0000000000006434 28403074 PMC5403071

[B253] Suhaimi M. S. (2017). The Use of Buprenorphine/naltrexone Combination Treatment in Attenuating Relapse to Morphine/methamphetamine Polydrug-Dependence in Mice. Master's thesis (Kuantan, Pahang: International Islamic University Malaysia).

[B254] Suzuki J. El-Haddad S. (2017). A Review: Fentanyl and Non-pharmaceutical Fentanyls. Drug Alcohol Depend 171, 107–116. 10.1016/j.drugalcdep.2016.11.033 28068563

[B255] Tan K. Davis J. P. Smith D. C. Yang W. (2020). Individual, Family, and School Correlates across Patterns of High School Poly-Substance Use. Subst. Use Misuse 55, 743–751. 10.1080/10826084.2019.1701035 31829078

[B256] Tata D. A. Yamamoto B. K. (2007). Interactions between Methamphetamine and Environmental Stress: Role of Oxidative Stress, Glutamate and Mitochondrial Dysfunction. Addiction 102, 49–60. 10.1111/j.1360-0443.2007.01770.x 17493053

[B257] Tatsuta T. Kitanaka N. Kitanaka J. Morita Y. Takemura M. (2007). Lack of Effect of Anticonvulsant Topiramate on Methamphetamine-Induced Stereotypy and Rewarding Property in Mice. Pharmacol. Biochem. Behav. 87, 48–55. 10.1016/j.pbb.2007.03.019 17482247

[B258] Teoh Bing Fei J. Yee A. Habil M. H. Danaee M. (2016). Effectiveness of Methadone Maintenance Therapy and Improvement in Quality of Life Following a Decade of Implementation. J. Subst. Abuse Treat. 69, 50–56. 10.1016/j.jsat.2016.07.006 27568510

[B259] The Lancet (2018). Opioids and Methamphetamine: a Tale of Two Crises. Lancet 391, 713. 10.1016/S0140-6736(18)30319-2 29486928

[B260] Thompson P. M. Hayashi K. M. Simon S. L. Geaga J. A. Hong M. S. Sui Y. (2004). Structural Abnormalities in the Brains of Human Subjects Who Use Methamphetamine. J. Neurosci. 24 (26), 6028–6036. 10.1523/jneurosci.0713-04.2004 15229250 PMC6729247

[B261] Tien L. T. Ho I. K. (2011). Involvement of Μ-Opioid Receptor in Methamphetamine-Induced Behavioral Sensitization. Curr. Neuropharmacol 9 (1), 215–218. 10.2174/157015911795016949 21886593 PMC3137186

[B262] Tiihonen J. Krupitsky E. Verbitskaya E. Blokhina E. Mamontova O. Föhr J. (2012). Naltrexone Implant for the Treatment of Polydrug Dependence: a Randomized Controlled Trial. Am. J. Psychiatry 169 (5), 531–536. 10.1176/appi.ajp.2011.11071121 22764364

[B263] Tolomeo S. Gray S. Matthews K. Steele J. D. Baldacchino A. (2016). Multifaceted Impairments in Impulsivity and Brain Structural Abnormalities in Opioid Dependence and Abstinence. Psychol. Med. 46 (13), 2841–2853. 10.1017/s0033291716001513 27452238

[B264] Tomczyk S. Isensee B. Hanewinkel R. (2016). Latent Classes of Polysubstance Use Among Adolescents-A Systematic Review. Drug Alcohol Depend 160, 12–29. 10.1016/j.drugalcdep.2015.11.035 26794683

[B265] Toubia T. Khalife T. (2019). The Endogenous Opioid System: Role and Dysfunction Caused by Opioid Therapy. Clin. Obstet. Gynecol. 62 (1), 3–10. 10.1097/grf.0000000000000409 30398979

[B266] Trivedi M. H. Walker R. Ling W. dela Cruz A. Sharma G. Carmody T. (2021). Bupropion and Naltrexone in Methamphetamine Use Disorder. N. Engl. J. Med. 384 (2), 140–153. 10.1056/NEJMoa2020214 33497547 PMC8111570

[B267] Trujillo K. A. Smith M. L. Guaderrama M. M. (2011). Powerful Behavioral Interactions between Methamphetamine and Morphine. Pharmacol. Biochem. Behav. 99 (3), 451–458. 10.1016/j.pbb.2011.04.014 21549146 PMC3197716

[B268] Tsui J. I. Mayfield J. Speaker E. C. Yakup S. Ries R. Funai H. (2020). Association between Methamphetamine Use and Retention Among Patients with Opioid Use Disorders Treated with Buprenorphine. J. Subst. Abuse Treat. 109, 80–85. 10.1016/j.jsat.2019.10.005 31810594

[B269] Tsukada H. Nishiyama S. Kakiuchi T. Ohba H. Sato K. Harada N. (1999). Is Synaptic Dopamine Concentration the Exclusive Factor Which Alters the *In Vivo* Binding of [11C]raclopride? PET Studies Combined with Microdialysis in Conscious Monkeys. Brain Res. 841 (1-2), 160–169. 10.1016/S0006-8993(99)01834-X 10546998

[B270] UN World Drug Report (2018). World Drug Report 2018. (E.18.XI.9). Available at: https://www.unodc.org/wdr2018/prelaunch/WDR18_Booklet_2_GLOBAL.pdf .

[B271] UN World Drug Report (2019). World Drug Report 2019. (E.19.XI.8). Available at: https://wdr.unodc.org/wdr2019/prelaunch/WDR19_Booklet_2_DRUG_DEMAND.pdf .

[B272] UN World Drug Report (2021). World Drug Report 2021. (E.21.XI.8). Available at: https://www.unodc.org/res/wdr2021/field/WDR21_Booklet_2.pdf .

[B273] Valente P. K. Bazzi A. R. Childs E. Salhaney P. Earlywine J. Olson J. (2020). Patterns, Contexts, and Motivations for Polysubstance Use Among People Who Inject Drugs in Non-urban Settings in the U.S. Northeast. Int. J. Drug Pol. 85, 102934. 10.1016/j.drugpo.2020.102934 PMC7770041 32911318

[B274] Vassoler F. M. Wright S. J. Byrnes E. M. (2016). Exposure to Opiates in Female Adolescents Alters Mu Opiate Receptor Expression and Increases the Rewarding Effects of Morphine in Future Offspring. Neuropharmacology 103, 112–121. 10.1016/j.neuropharm.2015.11.026 26700246 PMC4755844

[B275] Verster J. C. Scholey A. Dahl T. A. Iversen J. M. (2021). Functional Observation after Morphine Withdrawal: Effects of SJP-005. Psychopharmacology (Berl) 238 (6), 1449–1460. 10.1007/s00213-021-05771-5 33555386 PMC8139893

[B276] Vicknasingam B. Mazlan M. Schottenfeld R. S. Chawarski M. C. (2010). Injection of Buprenorphine and Buprenorphine/naloxone Tablets in Malaysia. Drug Alcohol Depend 111 (1-2), 44–49. 10.1016/j.drugalcdep.2010.03.014 20478668

[B277] Vijay A. Bazazi A. R. Yee I. Kamarulzaman A. Altice F. L. (2015). Treatment Readiness, Attitudes toward, and Experiences with Methadone and Buprenorphine Maintenance Therapy Among People Who Inject Drugs in Malaysia. J. Subst. Abuse Treat. 54, 29–36. 10.1016/j.jsat.2015.01.014 25841703 PMC4758679

[B278] Volkow N. D. Chang L. Wang G. J. Fowler J. S. Leonido-Yee M. Franceschi D. (2001). Association of Dopamine Transporter Reduction with Psychomotor Impairment in Methamphetamine Abusers. Am. J. Psychiatry 158 (3), 377–382. 10.1176/appi.ajp.158.3.377 11229977

[B279] Vuletic D. Dupont P. Robertson F. Warwick J. Zeevaart J. R. Stein D. J. (2018). Methamphetamine Dependence with and without Psychotic Symptoms: A Multi-Modal Brain Imaging Study. Neuroimage Clin. 20, 1157–1162. 10.1016/j.nicl.2018.10.023 30380522 PMC6205927

[B280] Walsh S. L. Preston K. L. Stitzer M. L. Cone E. J. Bigelow G. E. (1994). Clinical Pharmacology of Buprenorphine: Ceiling Effects at High Doses. Clin. Pharmacol. Ther. 55 (5), 569–580. 10.1038/clpt.1994.71 8181201

[B281] Wang G. J. Smith L. Volkow N. D. Telang F. Logan J. Tomasi D. (2012). Decreased Dopamine Activity Predicts Relapse in Methamphetamine Abusers. Mol. Psychiatry 17, 918–925. 10.1038/mp.2011.86 21747399 PMC3261322

[B282] Wang L. Zou F. Zhai T. Lei Y. Tan S. Jin X. (2016). Abnormal gray Matter Volume and Resting-State Functional Connectivity in Former Heroin-dependent Individuals Abstinent for Multiple Years. Addict. Biol. 21 (3), 646–656. 10.1111/adb.12228 25727574

[B283] Wang S. (2019). Historical Review: Opiate Addiction and Opioid Receptors. Cell Transpl. 28 (3), 233–238. 10.1177/0963689718811060 PMC6425114 30419763

[B284] Webster L. Hjelmström P. Sumner M. Gunderson E. W. (2016). Efficacy and Safety of a Sublingual Buprenorphine/naloxone Rapidly Dissolving Tablet for the Treatment of Adults with Opioid Dependence: a Randomized Trial. J. Addict. Dis. 35 (4), 325–338. 10.1080/10550887.2016.1195608 27267785

[B285] Willi T. S. Honer W. G. Thornton A. E. Gicas K. Procyshyn R. M. Vila-Rodriguez F. (2016). Factors Affecting Severity of Positive and Negative Symptoms of Psychosis in a Polysubstance Using Population with Psychostimulant Dependence. Psychiatry Res. 240, 336–342. 10.1016/j.psychres.2016.04.059 27138828

[B286] Willis E. Adams R. Keene J. (2019). If Everyone Is Doing it, it Must Be Safe: College Students' Development of Attitudes toward Poly-Substance Use. Subst. Use Misuse 54, 1886–1893. 10.1080/10826084.2019.1618334 31142176

[B287] Winkelman T. N. A. Chang V. W. Binswanger I. A. (2018). Health, Polysubstance Use, and Criminal justice Involvement Among Adults with Varying Levels of Opioid Use. JAMA Netw. Open 1, e180558. 10.1001/jamanetworkopen.2018.0558 30646016 PMC6324297

[B288] Wise R. A. Bozarth M. A. (1987). A Psychomotor Stimulant Theory of Addiction. Psychol. Rev. 94, 469–492. 10.1037/0033-295x.94.4.469 3317472

[B289] Wise R. A. (1978). Catecholamine Theories of Reward: a Critical Review. Brain Res. 152, 215–247. 10.1016/0006-8993(78)90253-6 354753

[B290] Withey S. L. Cao L. de Moura F. B. Cayetano K. R. Rohan M. L. Bergman J. (2022). Fentanyl-induced Changes in Brain Activity in Awake Nonhuman Primates at 9.4 Tesla. Brain Imaging Behav., 1–11. 10.1007/s11682-022-00639-4 35226333 PMC12412339

[B291] Yeh T. L. Chen K. C. Lin S. H. Lee I. H. Chen P. S. Yao W. J. (2012). Availability of Dopamine and Serotonin Transporters in Opioid-dependent Users-Aa Two-Isotope SPECT Study. Psychopharmacology (Berl) 220 (1), 55–64. 10.1007/s00213-011-2454-6 21881874

[B292] Yokell M. A. Zaller N. D. Green T. C. Rich J. D. (2011). Buprenorphine and Buprenorphine/naloxone Diversion, Misuse, and Illicit Use: an International Review. Curr. Drug Abuse Rev. 4 (1), 28–41. 10.2174/1874473711104010028 21466501 PMC3154701

[B293] Yonemitsu K. Sasao A. Mishima S. Ohtsu Y. Nishitani Y. (2016). A Fatal Poisoning Case by Intravenous Injection of "bath Salts" Containing Acetyl Fentanyl and 4-methoxy PV8. Forensic Sci. Int. 267, e6–e9. 10.1016/j.forsciint.2016.08.025 27591912

[B294] Zhang D. Zhang H. Jin G. Z. Zhang K. Zhen X. (2008). Single Dose of Morphine Produced a Prolonged Effect on Dopamine Neuron Activities. Mol. Pain 4, 57–8069. 10.1186/1744-8069-4-57 19014677 PMC2603002

[B295] Zhang J. Su H. Tao J. Xie Y. Sun Y. Li L. (2015). Relationship of Impulsivity and Depression during Early Methamphetamine Withdrawal in Han Chinese Population. Addict. Behav. 43, 7–10. 10.1016/j.addbeh.2014.10.032 25513754

[B296] Zhang L. Zou X. Xu Y. Medland N. Deng L. Liu Y. (2019). The Decade-Long Chinese Methadone Maintenance Therapy Yields Large Population and Economic Benefits for Drug Users in Reducing Harm, HIV and HCV Disease Burden. Front. Public Health 7, 327. 10.3389/fpubh.2019.00327 31781529 PMC6861367

[B297] Zhang R. Jiang G. Tian J. Qiu Y. Wen X. Zalesky A. (2016). Abnormal white Matter Structural Networks Characterize Heroin-dependent Individuals: a Network Analysis. Addict. Biol. 21 (3), 667–678. 10.1111/adb.12234 25740690

[B298] Zhang Y. Tian J. Yuan K. Liu P. Zhuo L. Qin W (2011). Distinct Resting-State Brain Activities in Heroin-dependent Individuals. Brain Res. 1402, 46–53. 10.1016/j.brainres.2011.05.054 21669407

[B299] Zhang Z. He L. Huang S. Fan L. Li Y. Li P. (2018). Alteration of Brain Structure with Long-Term Abstinence of Methamphetamine by Voxel-Based Morphometry. Front. Psychiatry 9, 722. 10.3389/fpsyt.2018.00722 30618890 PMC6306455

[B300] Zhang Z. Schulteis G. (2008). Withdrawal from Acute Morphine Dependence Is Accompanied by Increased Anxiety-like Behavior in the Elevated Plus Maze. Pharmacol. Biochem. Behav. 89 (3), 392–403. 10.1016/j.pbb.2008.01.013 18308382 PMC2323908

[B301] Zhao J. Kral A. H. Simpson K. A. Ceasar R. C. Wenger L. D. Kirkpatrick M. (2021). Factors Associated with Methamphetamine Withdrawal Symptoms Among People Who Inject Drugs. Drug Alcohol Depend 223 (2021), 108702. 10.1016/j.drugalcdep.2021.108702 33894459 PMC12994592

[B302] Zhou Y. Bendor J. Hofmann L. Randesi M. Ho A. Kreek M. J. (2006). Mu Opioid Receptor and Orexin/hypocretin mRNA Levels in the Lateral Hypothalamus and Striatum Are Enhanced by Morphine Withdrawal. J. Endocrinol. 191 (1), 137–145. 10.1677/joe.1.06960 17065397

[B303] Zhu L. Zhu J. Liu Y. Chen Y. Li Y. Huang L. Chen S. Li T. Dang Y. Chen T. (2015). Methamphetamine Induces Alterations in the Long Non-coding RNAs Expression Profile in the Nucleus Accumbens of the Mouse. BMC Neurosci. 16 (1), 18–13. 10.1186/s12868-015-0157-3 25884509 PMC4399149

[B304] Zorick T. Nestor L. Miotto K. Sugar C. Hellemann G. Scanlon G. Rawson R. London E. D. (2010). Withdrawal Symptoms in Abstinent Methamphetamine-dependent Subjects. Addiction 105 (10), 1809–1818. 10.1111/j.1360-0443.2010.03066.x 20840201 PMC3071736

[B305] Zuckermann A. M. E. Williams G. Battista K. de Groh M. Jiang Y. Leatherdale S. T. (2019). Trends of Poly-Substance Use Among Canadian Youth. Addict. Behav. Rep. 10, 100189. 10.1016/j.abrep.2019.100189 31193263 PMC6525276

[B306] Zuckermann A. M. E. Williams G. C. Battista K. Jiang Y. de Groh M. Leatherdale S. T. (2020). Prevalence and Correlates of Youth Poly-Substance Use in the COMPASS Study. Addict. Behav. 107, 106400. 10.1016/j.addbeh.2020.106400 32222564

